# Cryo-EM structure of *Saccharomyces cerevisiae* target of rapamycin complex 2

**DOI:** 10.1038/s41467-017-01862-0

**Published:** 2017-11-23

**Authors:** Manikandan Karuppasamy, Beata Kusmider, Taiana M. Oliveira, Christl Gaubitz, Manoel Prouteau, Robbie Loewith, Christiane Schaffitzel

**Affiliations:** 1 European Molecular Biology Laboratory, Grenoble Outstation, 71 Avenue des Martyrs, 38042 Grenoble, France; 2 Department of Molecular Biology, Institute of Genetics and Genomics of Geneva (iGE3), University of Geneva, 30 Quai Ernest Ansermet, CH1211 Geneva, Switzerland; 3 Swiss National Centre for Competence in Research (NCCR) in Chemical Biology, University of Geneva, 30 Quai Ernest-Ansermet, Bristol, CH1211 Geneva Switzerland; 4 School of Biochemistry, University of Bristol, University Walk, Bristol, BS8 1TD UK

## Abstract

The target of rapamycin (TOR) kinase assembles into two distinct multiprotein complexes, conserved across eukaryote evolution. In contrast to TOR complex 1 (TORC1), TORC2 kinase activity is not inhibited by the macrolide rapamycin. Here, we present the structure of *Saccharomyces cerevisiae* TORC2 determined by electron cryo-microscopy. TORC2 contains six subunits assembling into a 1.4 MDa rhombohedron. Tor2 and Lst8 form the common core of both TOR complexes. Avo3/Rictor is unique to TORC2, but interacts with the same HEAT repeats of Tor2 that are engaged by Kog1/Raptor in mammalian TORC1, explaining the mutual exclusivity of these two proteins. Density, which we conclude is Avo3, occludes the FKBP12-rapamycin-binding site of Tor2’s FRB domain rendering TORC2 rapamycin insensitive and recessing the kinase active site. Although mobile, Avo1/hSin1 further restricts access to the active site as its conserved-region-in-the-middle (CRIM) domain is positioned along an edge of the TORC2 active-site-cleft, consistent with a role for CRIM in substrate recruitment.

## Abstract (web summary)

Target of rapamycin (TOR) kinase operates within two distinct multiprotein complexes named TORC1 and TORC2. Here the authors report a cryo-EM structure of TORC2, establish its subunit organization, providing a rationale for TORC2’s rapamycin insensitivity and the mutually exclusive inclusion of Avo3/Rictor or Raptor within their respective TOR complex.

## Introduction

The target of rapamycin (*TOR*) genes were first discovered in an elegant genetic dissection of *Saccharomyces cerevisiae* mutants exhibiting resistance to rapamycin^1^, a macrolide produced by a soil bacterium from Easter Island^2^. This study also demonstrated that rapamycin requires a co-factor for toxicity, a 12 kDa proline isomerase named FKBP12 for FK506 (a macrolide similar to rapamycin)-binding protein. Subsequent to its discovery in yeast, a mammalian ortholog (*mTOR*) was described^3–5^ and indeed it is now appreciated that *TOR* is widely conserved in eukaryotes^6^. Unlike yeast, which encodes two *TOR* genes, *TOR1* and *TOR2*, higher eukaryotes encode only a single *TOR* gene. The amino acid sequence of Tor initially suggested that it would function as a lipid kinase^7,8^. However, Tor is a Ser/Thr protein kinase and the founding member of a family of related proteins including DNA-PKcs, SMG1, ATR and ATM, known as phosphatidyl-inositol-kinase-like kinases (PIKKs), which all resemble phosphatidyl-inositol kinases but are in fact Ser/Thr protein kinases^9^. Among the PIKKs, only Tor, at its FKBP12-rapamycin-binding domain (FRB) in the N-terminus of the kinase domain, can be bound, and thus inhibited, by a complex of rapamycin and FKBP12.

Biochemical purification of Tor1 and Tor2 from yeast demonstrated that Tor exists in two different multiprotein complexes named TORC1 and TORC2^10–12^. Like *TOR*, these complexes are widely conserved, and, in mammals, are known as mTORC1 and mTORC2^13–17^. Both complexes are heterodimeric and are 1.2 MDa and 1.4 MDa in size, respectively^18^. TORC1 (mTORC1) is composed of either Tor1 or Tor2 (mTOR), Kog1 (Raptor), Lst8 (mLst8/GβL) and Tco89 (no ortholog). Rapamycin-FKBP12 binding to the FRB of Tor in TORC1 inhibits kinase activity via steric occlusion of the active site^19^. In humans, abnormal mTORC1 activity is associated with a wide range of clinical presentations for which rapamycin or related compounds are often indicated^20^. These include cancer, loss of metabolic homoeostasis, immune dysfunction and ageing. Rapamycin has also been a useful tool for basic scientists to study TORC1 signalling. Indeed, the mechanisms by which upstream regulators (eg, nutrients, abiotic stress and growth factors) regulate TORC1, as well as the downstream targets by which TORC1 regulates its distal effectors (eg, lipid, nucleotide and protein synthesis/degradation) are becoming increasingly well understood^20–23^.

**Fig. 1:**
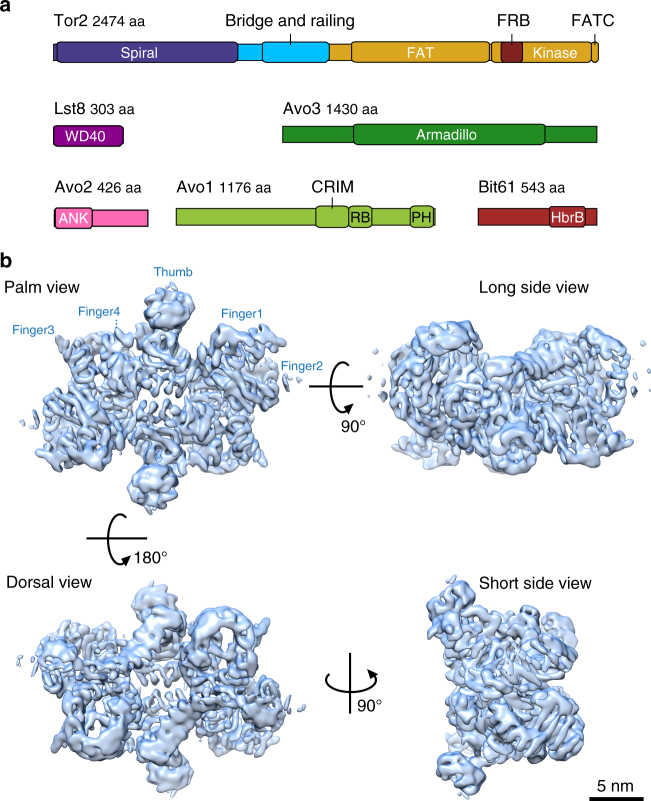
Overall architecture of *S*. *cerevisiae* TORC2. **a** Schematic representation of TORC2 proteins. Domains of known function and structural motifs are indicated. **b** Cryo-EM structure of TORC2 at an overall resolution of 7.9 Å (FSC criterion 0.143) is shown in a surface representation at 7.5 sigma contour level in four different views. In the palm view, TORC2 lobes are annotated as Thumb and Fingers (1–4) for one protomer. Scale bar = 5 nm. FAT stands for Frap, ATM, TRRAP domain; FRB, FKBP12-rapamycin-binding domain; FATC, FRAP, ATM and TRRAP, C-terminal domain; Armadillo, armadillo repeat domain; ANK, ankyrin repeat domain; CRIM, conserved-region-in-the-middle domain; RB, Ras-binding domain; PH, pleckstrin homology domain; HbrB, HbrB-like domain

TORC2 (mTORC2) is composed of Tor2 (mTOR), Lst8 (mLst8/GβL), Avo1 (hSin1), Avo2 (no ortholog), Avo3 (Rictor) and Bit61 or its paralog Bit2 (Protor1 or Protor2). The FRB domain of Tor2 has been suggested to be inaccessible in TORC2, which would explain why the kinase activity of this complex is insensitive to acute treatment with rapamycin^24^. Like mTORC1, mTORC2 dysregulation is implicated in cancer and metabolic perturbations^20,25^, but unlike mTORC1, specific small-molecule inhibitors of mTORC2 are not presently available. Consequently, the mechanisms of upstream regulators (eg, plasma membrane tension and growth factors) and downstream effectors (eg, endocytosis, lipid biosynthesis, genome stability, survival and proliferation) of TORC2 are relatively less well characterized^26–30^. Further insights into the TOR complexes have come from recent structural studies that have revealed the molecular architecture of TORC1. From N-terminus to C-terminus, Tor contains two blocks of HEAT (Huntingtin, EF3A, ATM, TOR) repeats, a block of TPR (tetratricopeptide) repeats known as the FAT (Frap, ATM, TRRAP) domain, and the kinase domain, which includes the FRB domain inserted in its N-terminal part and the FAT C-terminal (FATC) domain^19,31^ (Fig. 1a). Lst8 is composed entirely of WD40 repeats (Fig. 1a). A crystal structure of the FAT-FRB-Kinase-FATC domain of mTOR in complex with mLst8 demonstrated that the catalytic domain adopts a canonical protein kinase fold, cradled by the FAT domain^19^. Moreover, this study showed that binding of FKBP12-rapamycin to the FRB domain would further occlude access to an already deeply recessed active site.

More recently, combinations of electron cryo-microscopy (cryo-EM) and crystallographic studies, using proteins from mammals and thermo-tolerant yeast, have yielded additional insight into the organization of TORC1^19,32–34^. These structures revealed that the N-terminal HEAT blocks assemble into two α-helical solenoid super-structures, respectively, referred to as the “spiral”^33^ or “horn”^32^ and the “bridge” that is followed by an extended linker referred to as the “railing” (Fig. 1a). These solenoids form the bulk of the interface between the two protomers in TORC1 although the armadillo repeats of Raptor also contribute to this interaction^32,34^. The conserved N-terminus of Raptor is juxtaposed to the kinase active site, in agreement with its role in recruiting protein substrates^32^.

We provided preliminary insights into the molecular architecture of *S*. *cerevisiae* TORC2 using negative stain EM and crosslinking/mass spectrometry (XL/MS)^24^. This work defined a rhombohedral shape for TORC2, globally similar to, but slightly larger than mTORC1^35^. We found that deletion of the C-terminal 157 amino acids of Avo3 made the kinase activity of TORC2 sensitive to inhibition with rapamycin-FKBP12 and accordingly, the C-terminus of Avo3 was suggested to lie in proximity to the FRB domain of Tor2^28^. The PH domain located in the C-terminus of Avo1 was placed at the obtuse edge of the rhombohedron. Curiously, in the recent high-resolution mTORC1 structures, this region is clearly occupied by mLst8^32,34^.

Here, we describe the cryo-EM structure of *S*. *cerevisiae* TORC2 at 7.9 Å resolution. TORC2 adopts a twofold symmetric rhombohedral structure comprising two copies of each subunit. We reveal the subunit architecture of the complex, the topology of Lst8 and Tor2 in the complex and the interactions at the dimer interface. We find that Avo3 interacts with Tor2 in a manner analogous to the interaction of Raptor with mTOR in mTORC1, sterically excluding Kog1/Raptor incorporation in TORC2. Avo3 additionally occupies volume adjacent to the Tor2 FRB domain that prevents rapamycin-FKBP12 binding and thus renders TORC2 rapamycin insensitive and serves to further restrict access to the already recessed active site. Density that we attribute to Avo1 resolves poorly, suggesting that this subunit is highly mobile. In agreement with XL/MS experiments^24^, we propose that the PH domain of Avo1 appears to float above Lst8 and that its conserved-region-in-the-middle (CRIM) domain lines the active site pocket. We corroborate this localization by subunit localization experiments using negative stain EM. Our placement of the CRIM domain is consistent with its proposed role in substrate recruitment^36^.

## Results

### The architecture of TORC2

TORC2 was purified from a *S*. *cerevisiae* cell extract via protein-A affinity purification of Bit61-TAP from a Bit2 knockout strain (Supplementary Fig. 1a). After confirming the integrity of the purified complex by negative stain EM (Supplementary Fig. 1b), the same preparation was used for cryo-EM grid preparation. Cryo-EM data were collected on a Titan Krios electron microscope with a direct electron detector (DED) (Supplementary Fig. 2a). The initial data set comprised 111,022 particles of which 16,190 were included in the final reconstruction (Supplementary Fig. 2b, c). Subsequently, a second data set of 71,519 particles was collected and, after multiple rounds of sorting at two-dimensional (2D) and three-dimensional (3D) levels, an additional 10,663 particles were included in the final TORC2 reconstruction (26,853 particles in total). The low-resolution TORC2 negative stain reconstruction^24^ (Supplementary Fig. 1c) was used as an initial 3D model, yielding a final reconstruction of the TORC2 rhombohedron (Fig. 1b) with an average resolution of 7.9 Å (Supplementary Fig. 2d). The central part of TORC2 possesses a narrow cavity with surrounding density, which is well resolved up to 5 Å. In contrast, the peripheral portions of the complex are resolved to a resolution of ~10–12 Å (Supplementary Fig. 2e) suggesting that they are relatively more dynamic. As described below, we fitted available atomic models for Tor2 and Lst8 into this cryo-EM map. Subsequently, we placed models of the ankyrin repeats of Avo2, and the armadillo-like helical domain of Avo3. Finally, we propose localizations for the PH and CRIM domains of Avo1, which given their low resolution, appear to be highly mobile.

### Tor-Lst8 are similarly organized in TORC2 and mTORC1

**Fig. 2:**
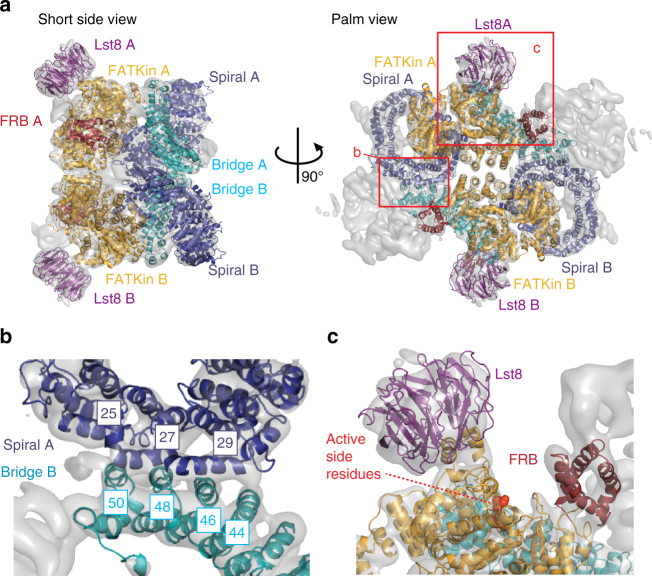
Fitting of *S*. *cerevisiae* Tor2-Lst8 dimer model into the TORC2 map. **a** Tor2-Lst8 model fitted into the cryo-EM density of TORC2 (transparent grey) shown from the short side view (left) and the palm view (right). The protomers are labelled A and B. Red boxes highlight the areas shown in a close-up view in **b**, **c**. **b** Close-up of the dimer interface formed by the spiral (purple) of one Tor2 molecule and the bridge (cyan) of the second Tor2. Helices Nα25, Nα27 and Nα29 (spiral) and Nα44, Nα46, Nα48 and Nα50 (bridge) are involved in interface contacts. **c** Close-up of the Tor2 active site. Active site residues of the kinase are highlighted as red spheres. The FRB domain is depicted in dark red, the FAT/kinase domain (FATKin) in orange-yellow and Lst8 in purple

The well-resolved helical secondary structure elements of the centre of the complex, visible as tubes in the map contoured at 7.5 sigma level (Supplementary Fig. 2e), facilitated the fitting of a homology model of Tor2-Lst8 into the cryo-EM structure (Supplementary Movie 1). This *S*. *cerevisiae* model was built using the structure of the thermo-tolerant yeast *Kluyveromyces marxianus* (*Km*) Tor-Lst8 dimer^33^ (Fig. 2a). In agreement with previous structures^19,32–34^, Lst8 is bound to the catalytic kinase domain and positioned at the obtuse angle of the rhombohedron (Fig. 2a), clearly sticking out from the central part of the complex. Lst8 forms two contacts to the kinase domain; the major connection involves the Lst8-binding element (LBE)^19^ and a minor connection is formed by the Lst8-binding loop (LBL) (Supplementary Fig. 3). Both connections were also present in the *Km* Tor-Lst8 dimer^33^.

Overall, the conformation of *S*. *cerevisiae* Tor2 (*Sc* Tor2) in TORC2 is very similar to the Tor conformation in the *Km* Tor-Lst8 dimer structure^33^, (Supplementary Fig. 4a, b). After fitting, the average root-mean-square deviation for *Sc* Tor2, modelled from *Km* Tor, is 3 Å. C-alpha deviations of more than 4 Å from the *Km* Tor conformation are observed in the N-terminal residues of Tor2 (81–450) corresponding to part of the spiral domain and residues in the bridge domain (Supplementary Fig. 4a, b), indicating that there are conformational changes in these Tor2 domains upon assembly of TORC2. Moreover, the spiral and bridge domains of Tor2 form the majority of the protomer-protomer interface in TORC2, the Tor-Lst8 complex and also in mTORC1^33,34^. Specifically, in *Sc* TORC2, the dimer interface is mediated by four contacts between helices Nα25, 27, 29 of the spiral of Tor2 in one protomer, and helices Nα44, 46, 48, 50 of the bridge of Tor2 in the second protomer (Fig. 2b).

The overall shape and dimensions of TORC2 and mTORC1^32,34^ are very similar (Supplementary Fig. 5). However, the central cavity in TORC2 is significantly smaller and an interaction of the Tor2 molecules is observed in the centre of the complex. This is due to the fact that the Tor2 FAT domains, which cradle the kinase domain on one side and line the longer sides of the cavity on the other, are more closely packed in TORC2. Consequently, the distance between the Lst8 centre-of-masses is reduced to 158 Å in TORC2 compared to 172 Å in the mTORC1 structures.

As in mTORC1, and described further below, the active site in TORC2 is also deeply recessed, situated in the crevasse between the Thumb, formed by Lst8 and Finger1 (Fig. 2c, Supplementary Fig. 1c).

### Placement of TORC2-specific subunits

Beyond crystal structures of the 13 kDa pleckstrin homology (PH) domains of *S*. *cerevisiae* and human Avo1/hSin1^37^ and an nuclear magnetic resonance spectroscopy (NMRs) structure of the 16 kDa CRIM domain of the *Schizosaccharomyces pombe* Avo1 ortholog, Sin1^36,38^, high-resolution structures of TORC2-specific subunits are lacking.

**Fig. 3:**
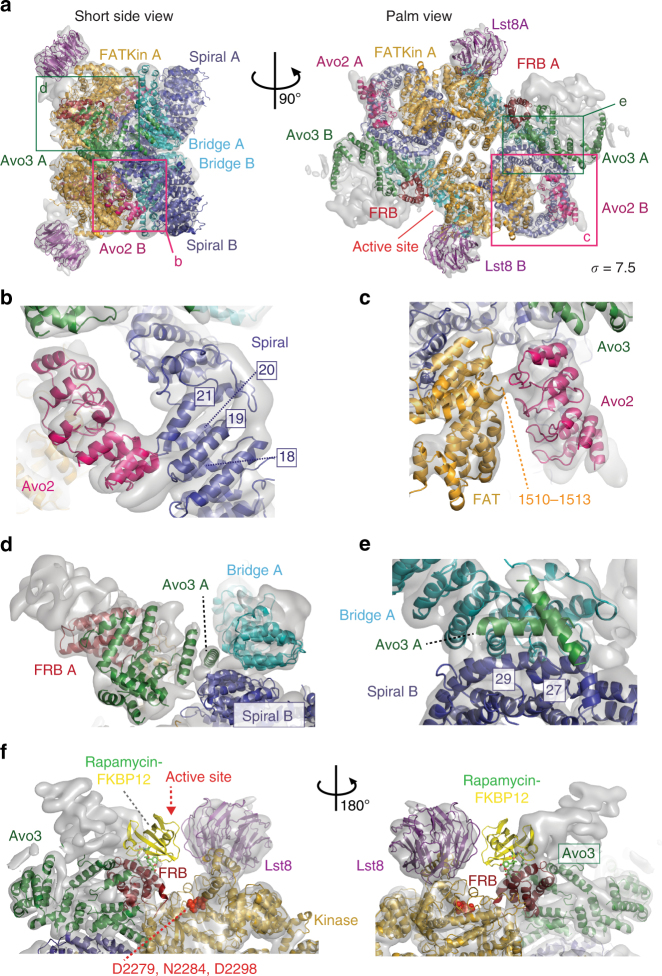
Fitting of Avo2 ankyrin repeats and Avo3 helices into TORC2. **a** TORC2 fitted with models for Avo2 ankyrin repeats (magenta), Avo3 helices (dark-green) and Tor2-Lst8 (colour coding as in Fig. 2). Boxes highlight the areas shown as close-up views in **b**–**e**. **b** Avo2 contacts loops formed by helices Nα18-Nα19 and Nα20-Nα21 of the Tor2 spiral (dark-blue). **c** Avo2 contacts FAT domain residues 1510–1513 (orange-yellow) of Tor2. **d** Two helices of Avo3 which likely are part of the armadillo-like helical domain contribute to the dimer interface by binding the Tor2 spiral of the other protomer. **e** Avo3 contacts helixes Nα27 and Nα29 of the adjacent Tor2 spiral. **f** Superimposition of the crystal structure of the human FKBP12-rapamycin-FRB complex (pdb ID: 1NSG) onto the Tor2 FRB domain, showing a clash between FKBP12 and Avo3. FKBP12 is shown in yellow and rapamycin in a stick representation. The transparent surface map is contoured at 7.5 sigma level

We attributed the remaining density of TORC2 building on previous XL-MS and EM subunit localization experiments^24^. These suggested that: Avo1 is located in the Thumb, Avo3 occupies the central part of the complex including regions close to the FRB domain; Bit61 forms the acute angle of the rhombohedron, which corresponds to Finger2 in the negative stain EM reconstruction (cf. Supplementary Fig. 1c) and Avo2 occupies Finger3, adjacent to Avo3 (Fig. 3a).

Avo2: The N-terminal portion of Avo2 is predicted to contain five ankyrin repeats (residues 4–171). We built a homology model for Avo2^39^ (Supplementary Table 1) and placed it into the density corresponding to Finger3, which shows clear alpha-helical features (Fig. 3b, c, Supplementary Table 2, Supplementary Movie 1). In this position, Avo2 forms one contact with the Tor2 spiral at the loops between helices Nα18 and 19 and between Nα20 and 21 (Fig. 3b) and a second contact with the Tor2 FAT domain (residues 1510–1513) (Fig. 3c).

Avo3: Avo3 comprises an armadillo-like helical domain that could be crosslinked to the HEAT, FAT and kinase domains of Tor2^24^. Accordingly, in the cryo-EM structure, we attribute the density in the central part of the complex, next to the Tor2 proteins, to Avo3. We built a partial Avo3 model fitting the well-resolved tube-shaped helices in the map (Fig. 3a, d, Supplementary Tables 1 and 2, Supplementary Movie 1). This central position of Avo3 and the extensive interactions between Avo3 and Tor2 suggest a role for Avo3 in forming and stabilizing the dimer interface. This is consistent with biochemical studies in which TORC2 integrity was found to depend upon Avo3^18^. In our model, Avo3 contacts the bridge of Tor2 (helices Nα46,48,50) in the same protomer and the spiral of Tor2 (helices Nα27 and Nα29) in the other protomer (Fig. 3d, e). Notably, in human mTORC1 the central armadillo domain of Raptor similarly contacts the bridge of mTOR in one protomer and the spiral of mTOR in the second protomer^34^ (Supplementary Fig. 6). In conclusion, Avo3 and Raptor occupy the same region of the interface formed by the Tor2/mTOR HEAT domains, committing the complexes to TORC2 and TORC1 assembly, respectively. This provides a structural basis for the prior biochemical studies that demonstrated that Avo3 and Kog1/Raptor binding to Tor-Lst8 is mutually exclusive^10,14,17^.

Strikingly, significant density is positioned above and next to the Tor2 FRB domain (Fig. 3f). Indeed, when superimposing the FKBP12-rapamycin-FRB structure^40^ onto the FRB in the fitted Tor2-Lst8 model, it becomes apparent that the rapamycin-binding site of FRB is not accessible in TORC2 (Fig. 3f). Due to the lack of a high-resolution Avo3 structure, we are unable to unambiguously assign this density surrounding the FRB domain. However, we found that deletion of the C-terminal 157 amino acids of Avo3 restores access of FKBP12-rapamycin to the FRB domain of Tor2^24^. Therefore, it is reasonable to assume that it is the C-terminal part of Avo3 that contacts the rapamycin-binding site of the FRB domain in the cryo-EM structure. The extensive contacts between Avo3 and the FRB domain additionally cause the TORC2 active site to be deeply recessed, even more than the active site of mTORC1 (Fig. 3f; Supplementary Fig. 6). This elongation of the active site and the extensive contacts of Avo3 with the FRB domain are likely to affect substrate selection.

**Fig. 4:**
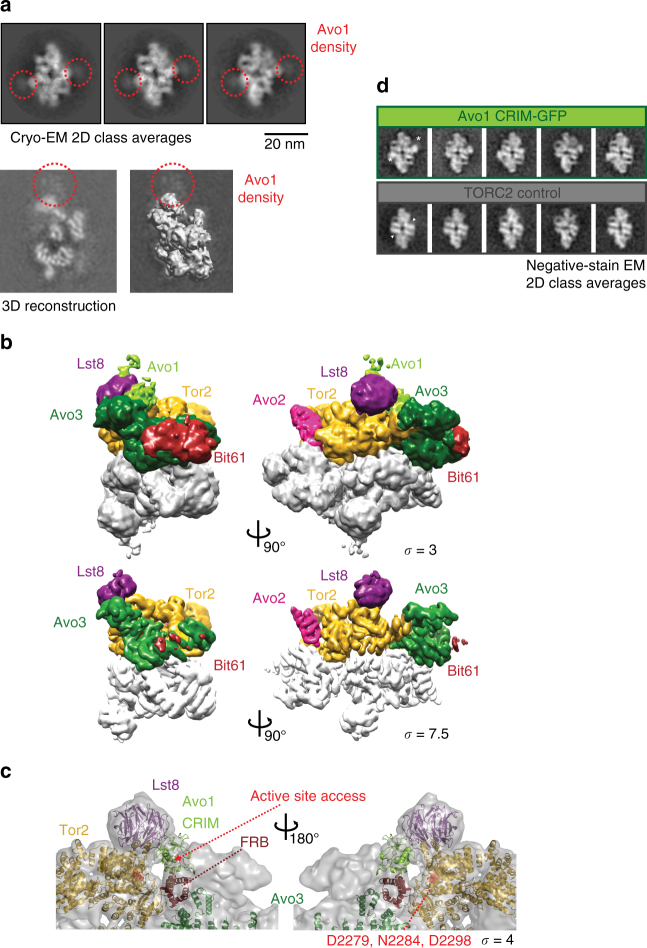
Avo1 is positioned next to Lst8 in TORC2. **a** Selected cryo-EM reference-free 2D class averages show a cloud of EM density (highlighted in red) next to the Thumb of TORC2. The additional density is attributed to Avo1 in line with XL-MS and EM localisation experiments^24^. Below: a slice of the reconstructed TORC2 volume is shown without (left) and with the final map contoured at 3.5 sigma level (right). Extra density attributed to Avo1, which extends the Thumb is highlighted (red circle). **b** Surface representations of the TORC2 map in a short side view (left) and the palm view (right). Above: TORC2 shown at 3 sigma; below: same views displayed at 7.5 sigma contour level. **c** The Avo1 CRIM homology model (yellow-green) fits into the additional density near the Tor2 active site. The domain contacts the rim of the Lst8 β propeller (purple) and the FRB-Avo3 interface. The map (transparent grey) is displayed at 4 sigma. The atoms of the active site residues (Asp2279, Asn2284 and Asp2298) are shown as red spheres. **d** Insertion of GFP into Avo1 after residue 794, immediately C-terminal to the CRIM domain (residues 647–792), generates new density inside the active site cleft of TORC2 in negative stain 2D class averages. New density is highlighted by white stars in the panel above; the active site of TORC2 is marked by white triangles below

Avo1: Previous data from negative stain EM suggested that the C-terminal PH domain of Avo1 is localized to the Thumb of TORC2^24^. However, in the high-resolution cryo-EM structure, the density forming the Thumb is clearly occupied by the WD40 domain of Lst8 (Fig. 2). We found an explanation for this apparent discrepancy: closer inspection of the reference-free cryo-EM 2D class averages reveals a halo of density around the Thumb/Lst8 region (Fig. 4a). This suggests that an additional protein, Avo1, is localized to this region, but the corresponding protein is very flexible and therefore poorly resolved. Localisation of Avo1 next to the Thumb is supported by XL/MS experiments^24^, which yielded several specific crosslinks between the middle part of Avo1 (adjacent to the CRIM domain) and Lst8 (Supplementary Movie 2). Consistently, we observe additional density in the same regions in projections of the TORC2 cryo-EM map as well (Fig. 4a). Attempts to improve the resolution of the density in this region of the map by focused classification and refinement were unsuccessful. Based on present observations and our previous localization efforts with negative stain EM involving TORC2 preparations in which the PH domain was either deleted or C-terminally tagged and labelled with an antibody^24^, we propose that the density on top of Lst8 corresponds to Avo1, including its PH domain that tethers TORC2 to the membrane^41^.

The apparent flexibility of the PH domain prompted us to further analyse an additional, less-resolved density next to Lst8: decreasing the contour level showed extra density present along the Thumb, lining the edge of the active-site-cleft (Fig. 4b). Specifically, this low-resolution density stretches from the Tor2 FRB domain/Avo3 lobe to the rim of the Lst8 β propeller and then occupies part of the face of the β propeller (Fig. 4b). At a contour level of 3 sigma, it is possible to fit the NMR structure of CRIM^36^ into this region (Fig. 4c). Consistent with this placement, we found that insertion of green fluorescent protein (GFP) into Avo1 immediately after the CRIM creates new density inside the active site cleft, adjacent to the Thumb (Fig. 4d). Although we cannot rule out that this new density represents a displacement of a native TORC2 subunit, collectively, these observations suggest that Avo1 localizes to the length and the tip of the Thumb, in proximity to the active site cleft, but adopts multiple conformations limiting the resolution of this region. Compared to mTORC1, Avo1, together with Avo3, significantly reshapes the active-site-cleft of TORC2 and further restricts the access of substrates (Fig. 4c).

## Discussion

**Fig. 5:**
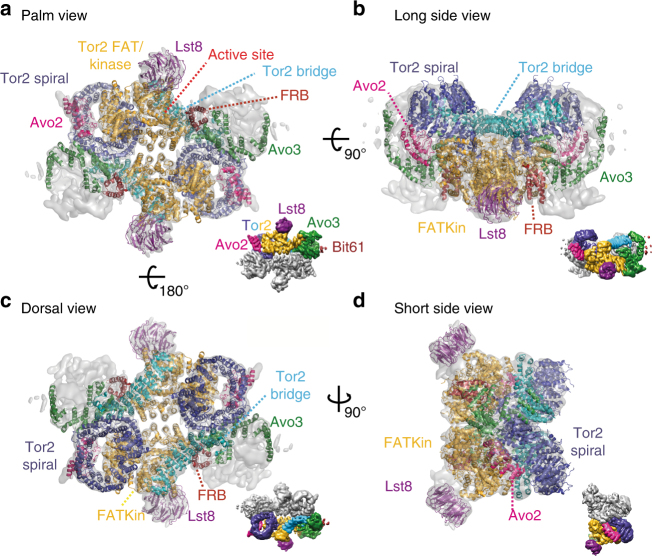
Atomic model of *S*. *cerevisiae* TORC2. The atomic model is complete for Tor2 (spiral in blue, bridge in cyan, FAT and kinase domain in orange-yellow and FRB in dark red) and for Lst8 (purple). In addition, the N-terminal five ankyrin repeats of Avo2 (magenta) as well as 17 individual alpha helices belonging to Avo3 (shown in green) are modelled. For each view **a**–**d**, a surface representation of the map is shown as inset with one of each subunit coloured as in the atomic model

Here, we describe the cryo-EM structure of TORC2 at an overall resolution of 7.9 Å. Into this structure, we could unambiguously place Tor2, Lst8, the ankyrin repeats of Avo2 and parts of Avo3 (Fig. 5). Tor2-Lst8 adopts a very similar structure in TORC2 and mTORC1, indicating a conserved core in both complexes. Compared to mTORC1, however, the kinase domains of Tor2 in TORC2 are closer to each other, which leads to a more compact complex with a smaller central cavity (Supplementary Fig. 5). As in mTORC1, the protomer-protomer interface of TORC2 is mediated largely by the HEAT repeats of Tor2 (Fig. 2). This interface is further stabilized by the armadillo-like helical domain of Avo3 (Fig. 3d, e) and by additional, weaker contacts formed by the helical repeats of the FAT domain lining the cavity in the middle of the complex (Fig. 5a; Supplementary Fig. 5). Importantly, Avo3 in TORC2 and Raptor in mTORC1, respectively, bind to exactly the same regions of the HEAT domains of the Tor kinase (Supplementary Fig. 6b, c). Thus, we propose that discrimination occurs at this stage in the molecular assembly of the complexes as incorporation of Raptor/Kog1 and Avo3 is mutually exclusive, priming the formation of TORC1 or TORC2.

As we move out from the centre of TORC2 that is resolved up to 5 Å, the resolution drops (Supplementary Fig. 2e), indicating flexibility of the peripherally localized TORC2 subunits. For example, despite the fact that we purified TORC2 via tagged Bit61 from cells lacking Bit2, the density for the Bit61 subunit is not well-defined, and beyond a general localization to Finger2, we cannot conclude more. Although the ankyrin repeats of Avo2 are placed with high confidence (Figs. 3b and 5), the localization to Finger3, distal from the kinase active site, does not readily suggest the function of this other, non-essential TORC2 subunit.

Avo1 that is an essential TORC2 subunit also appears to be highly mobile: specifically, in both reference-free cryo-EM 2D class averages and in projections of the TORC2 cryo-EM map, we observe a halo of density around the Thumb/Lst8 (Fig. 4a). Antibody labelling of the Avo1 PH domain, acquisition of a negative stain EM data from TORC2 lacking the Avo1 PH domain, GFP-tagging of the CRIM (Fig. 4d) and XL/MS data^24^ all support the notion that the PH and CRIM domains of Avo1 localized to the vicinity of the Thumb of TORC2, which in the cryo-EM structure is occupied by Lst8 (Fig. 5, Supplementary Movie 2). Although we cannot exclude that its flexibility is a consequence of the preparation method that requires detergents, it is interesting to consider that Avo1’s mobility might be required for its function in membrane binding or even membrane-tension sensing^26^.

The notion that Avo1 might be mobile relative to the core of TORC2 prompted us to look for additional, low-resolution density in our 2D class averages and to analyse the map at lower contour levels. This led to the discovery of additional density adjacent to the β propeller of Lst8, along an edge of the active-site cleft, which can accommodate the CRIM domain of Avo1 (Fig. 4c). Supporting this localization, residues adjacent to the CRIM domain crosslinked to Lst8^24^ and we found that insertion of a GFP module immediately after the CRIM sequence of Avo1 created new density inside the active site cleft (Fig. 4d). Placement of the CRIM domain in such close proximity to the active site is consistent with the recently proposed role of this domain in binding and recruitment of substrate to TORC2^36^. Thus, the CRIM domain could be functionally analogous to the meta-caspase-like domain of Raptor, which, in mTORC1, is proposed to be similarly involved in substrate recruitment^32^.

Notably, our present TORC2 cryo-EM structure demonstrates that Avo3 is localized in proximity to the FRB domain of Tor2 and sterically interferes with FKBP12-rapamycin binding. This is the molecular basis of TORC2’s insensitivity to rapamycin. Accordingly, deletion of the C-terminal part of Avo3 can render TORC2 rapamycin sensitive^24^. By binding to the FRB domain, Avo3 severely recesses the active site cleft and restricts substrate access to the active site. This notion is consistent with work from the Pavletich lab^19^ wherein it was proposed that the regulation of Tor kinase activity is mediated largely by controlling access of substrates to the active site cleft. Yang et al.^19^ also proposed that the FRB serves as a docking site for mTORC1 substrates. Our data suggest that this cannot be the case in TORC2 as the FRB domain is occluded by Avo3. This conclusion also calls into question previous proposals that mTORC2 activity is regulated through binding of phosphatidic acid to the FRB domain of mTOR^24,42,43^.

Taken together, the kinase active site cleft of TORC2 is surprisingly different from *Km* Tor-Lst8 and mTORC1. TORC2’s active site is shaped by Avo1 and Avo3, which both restrict access to the active-site cleft. These TORC2 subunits are thus perfectly positioned to mediate and control selection and access of TORC2 substrates.

## Methods

### Purification of *S*. *cerevisiae* TORC2

TORC2 was prepared through pull-down of TAP-tagged Bit61 from yeast extracts lacking Bit2^24^. Bit61-TAP expressing strains were grown in synthetic complete media to an equivalent optical density at 600 nm of 5.0. Cells were pelleted, snap frozen in liquid nitrogen and pulverized using Retsch MixerMill 400 or manually with a mortar and pestle. The pellet was resuspended in 1.5 volumes of extraction buffer (50 mM HEPES-KOH, pH 7.5, 5 mM CHAPS, 300 mM KCl, 0.5 mM DTT, complete protease inhibitor cocktail (Roche; 1 tablet per 50 ml) and 1 mM PMSF). After centrifugation, TORC2 was purified from the supernatant by ProteinA affinity purification using IgG-coated dynabeads (M270 Epoxy, Invitrogen). After washing (50 mM HEPES pH 7.5, 0.2 mM CHAPS, 300 mM KCl, 0.5 mM DTT), TORC2 was released from the beads by incubation with TEV protease (1 h at 18 °C).

### Cryo-EM grid preparation

Holey carbon grids (Quantifoil, type R2/2 300 mesh) were coated with a thin continuous carbon foil on top. Glow-discharged grids were incubated onto drops of 10–15 μl of TORC2 at 4 °C in order to increase the particle concentration on the grid. After 10 min incubation, the grids were blotted for 2–3 s at 4 °C and 100% relative humidity inside a Vitrobot Mark IV chamber (FEI, Eindhoven) and subsequently plunge-frozen in liquid ethane.

### Data collection and image processing

Single-particle cryo-EM data were collected using a FEI TITAN Krios microscope operated at 300 kV at EMBL Heidelberg. The data were recorded on a FEI Falcon II DED using automated data acquisition software (FEI EPU). A total of 4189 dose-fractionated movies each containing 40 frames with an accumulated total dose of 50 e/Å^2^ were recorded at a nominal magnification of ×59,000 corresponding to a pixel size of 1.38 Å and a total exposure time of 2.3 s. Images were acquired with a defocus range of −2 μm to −4 μm in steps of 0.1 μm. A second data set was collected from the same microscope using a K2 Summit detector (Gatan) with an energy filter. A total of 2847 dose-fractionated movies each containing 40 frames (0.5 s per frame) with a accumulated total dose of 47 e/Å^2^ were recorded semi-automatically using SerialEM^44^ at a nominal magnification of ×105,000 in super-resolution mode. Images were recorded with a defocus range of −1.5 μm to −3.5 μm. Subsequently, cropping in Fourier space resulted in a pixel size of 1.35 Å.

The movie frames from the Falcon II DED were aligned, dose-filtered and summed in Unblur1.0.2^45^. The individual movie frames from the K2 Summit detector were gain-corrected, aligned with the “patch” option, dose-weighted and summed using the MotionCor2 program^46^. The CTF parameters for both data sets were estimated from CTFFIND4^47^, integrated in Relion1.4^48^.

Automated particle picking was carried out in e2boxer.py from EMAN2^49^ with Gauss option with the box size of 320 pixels. All automatically picked particles were subjected to two rounds of 2D classification in Relion resulting in a pool of 111,196 good quality particles for data set 1 (from Falcon II). The TORC2 negative stain reconstruction (EMDB: 2990; ref. ^24^) was filtered to 60 Å and used as initial reference for 3D classification. At every step of classification, the data were grouped into two volumes (Supplementary Fig. 2). After three rounds of successive 3D classification with *K* = 2, a stable class with 16,190 particles yielded a well-defined volume (Supplementary Fig. 2). The second data set (from K2 Summit) consisting of 71,519 particles was sorted according to the same protocol and gave rise to additional 10,663 particles. The two data sets were combined after rescaling the second data set to a pixel size of 1.38 Å. The volume was further refined with a molecular shape mask (5 pixel Gaussian fall-off) and post-processed in Relion^48^ with C2 symmetry imposed. The resolution of the final volume was determined to be 7.9 Å based on the Fourier shell correlation (FSC) = 0.143 criterion^50^ (Supplementary Fig. 2d). During post-processing, the volume was corrected for the modulation transfer function of the detector and the effect of mask was checked by phase randomization^51^. The final map was sharpened with a B-factor value of −130 Å^2^. The local resolution analysis of the map was carried out using the program ResMap^52^.

### Focused classification and refinement of Avo1 density

The final, combined set of 26,853 particles was used for 3D classification without any alignment to two classes based on the molecular shape mask covering only the Lst8 and Avo1 part of the map. The classification converged with one volume populated with 94.6% of the particles and a featureless second volume from the residual data. Three-dimensional auto refinement using the 94.6% particles of first volume did not yield an improved map. Second, focused 3D refinement alone was performed with the mask described above. This also did not improve the density in this part of the map. This may be because the region of interest is very small compared to the total volume of the map and has a poorer signal. In addition, Avo1 appears to be highly flexible.

### Model building and generation of atomic model

The atomic model derived from the 6.1 Å map of the *Km* Tor–Lst8 complex^33^ was used as a template structure to obtain the homology model for *S*. *cerevisiae* Tor2. Homology modelling was performed in the Phyre2 homology modelling server^53^. The model was adjusted to fit into the map manually using COOT^54^ with the TORC2 map contoured at 7.5 sigma where individual helices are clearly visible as tubes (Supplementary Fig. 2 and Fig. 2). The remaining unfilled density was examined to fit other subunits (Avo1, Avo2 and Avo3). The secondary structure prediction of Avo1 indicates that this TORC subunit is largely unstructured, with the exception of the 13 kDa PH domain (aa 1076–1176; 3ulb; ref. ^37^) and the 16-kDa CRIM domain (aa 638–786; 2rvk; ref. ^36^). SwissModel^55^ was used to build a homology model for Avo1_CRIM based on the NMR structure of the Sin1 CRIM domain from fission yeast (2rvk; ref. ^36^). The Avo1_CRIM model (residues 647–792) fitted into the density between the Tor2 FRB domain and Lst8 (at 3.5 sigma level; cc of 0.87 in Chimera^56^). The N-terminal part of Avo2 has been predicted to contains five ankyrin repeats (residues 4–171)^39^. The homology model of the N-terminal part of Avo2 (aa 4–163) was generated in Phyre2 using the pdb structure 1n11 (chain A) as template. The Avo2 model fits well into the density previously assigned to Avo2^24^ connecting the Tor2 spiral domain on one side and the Tor2 C-terminal domain (cc of 0.94). For Avo3, a set of 17 helices of average length of 20 amino acids could be modelled into EM density contoured at 7.5 sigma. Secondary structure prediction suggests that the middle part of Avo3 contains an armadillo-like helical domain. Accordingly, we assign these alpha helices to the Avo3 armadillo-like domain. Moreover, the localization of these helices agrees with the predicted central position of Avo3 deduced from XL-MS and from negative stain EM domain localization experiments^24^. The partial model of TORC2 was refined against the density in real_space using the “phenix.real_space_refine” program^57^. The cross-correlation values for the docked atomic coordinates into the corresponding subunit EM map are listed in Supplementary Table 2 and the overall fitting was assessed by calculating the FSC between the atomic model and the corresponding parts of the map and the half maps using REFMAC^58^ (Supplementary Fig. 7). The refined model was used for all the interpretation. The figures were prepared in Chimera^56^ and PyMol (http://www.pymol.org/) (DeLano Scientific, San Carlos, CA, USA).

### Data availability

The cryo-EM map is available from the Electron Microscopy Data Band with accession code EMD-3896. The partial atomic model has been deposited in the Protein Data Bank with the accession code 6EMK. The data that support the findings of this study are available from the corresponding author upon request.

## Electronic supplementary material

Supplementary Information

Peer Review File

Description of Additional Supplementary Files

Supplementary Movie 1

Supplementary Movie 2
