# Supplementary material for: Cryo-EM structure of Saccharomyces cerevisiae target of rapamycin complex 2
Source: Nat Commun. 2017 Nov 23;8:1729. doi: 10.1038/s41467-017-01862-0 (PMC5700991; doi:10.1038/s41467-017-01862-0)
Supplement: Supplementary file 1 — Supplementary Information [file 41467_2017_1862_MOESM1_ESM.pdf]

Supplementary Figures and Tables

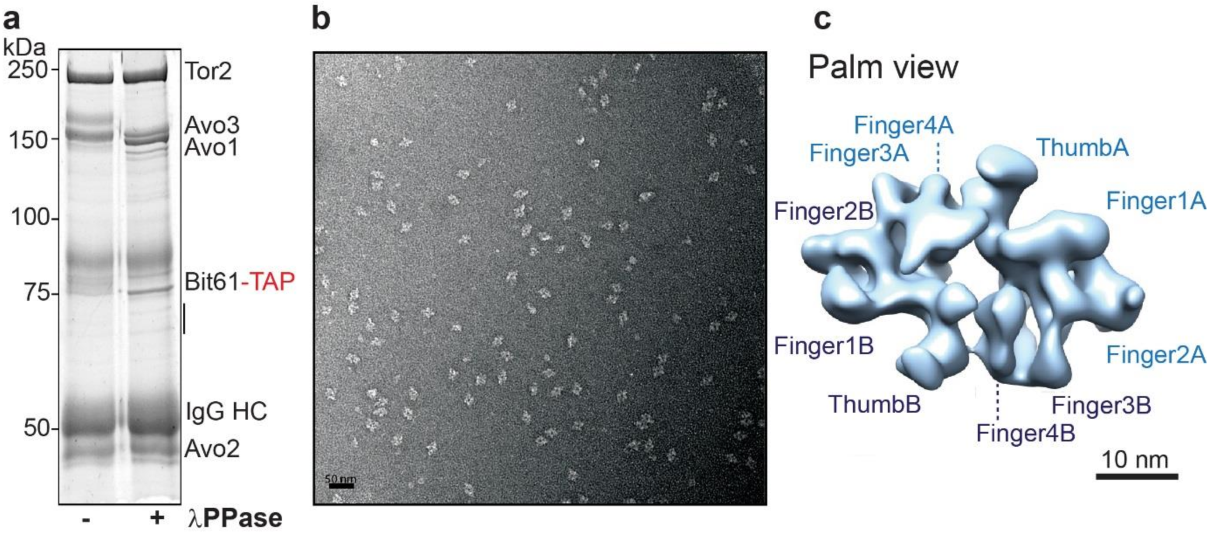

Supplementary Figure 1. TORC2 sample preparation and negative stain EM. a Sypro Ruby-stained SDS PAGE gel of TORC2 after protein-A affinity purification via TAP-tagged Bit61 from cell extracts lacking Bit2. Phosphorylation of TORC2 subunits Avo1 and Bit61 is detected by λ phosphatase treatment. Mass spectrometry additionally detected the presence of heat shock proteins SSA1 (69.6 kDa), SSA2 (69.4 kDa), SSB1 (66.6 kDa) and SSB2 (66.6 kDa), marked with a vertical black line. IgG antibody bands (partially reduced) are labelled. **b** Negative stain micrograph of purified TORC2. Scale bar corresponds to 50 nm. **c** Negative stain EM reconstruction showing the general architecture of TORC2 and annotation of the lobes as Thumb and Fingers (1 to 4) (adapted from Ref. 1). The interface of the protomers (labelled A and B) is formed between Fingers 2 and 3.

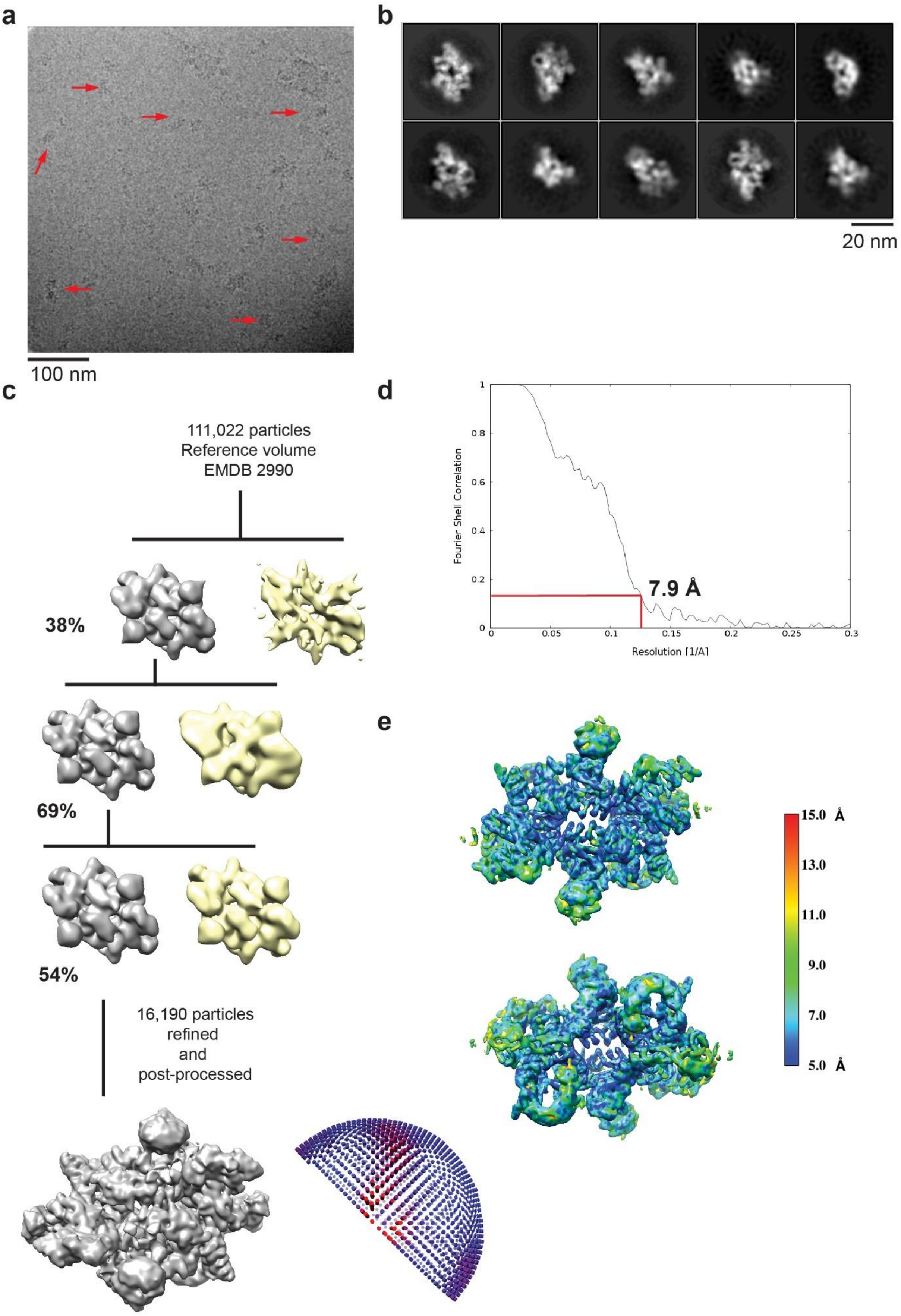
a
100 nm
b
20 nm
c
111,022 particles
Reference volume
EMDB 2990
38%
69%
54%
16,190 particles
refined
and
post-processed
d
Fourier Shell Correlation
Resolution [1/A]
7.9 Å
e
15.0 Å
13.0
11.0
9.0
7.0
5.0 Å

Supplementary Figure 2. Single-particle cryo-EM data processing of TORC2. a A representative micrograph with TORC2 particles highlighted by red arrows. **b** Reference-free 2D class averages. **c** Scheme for 3D classification of the first cryo-EM data set (from Falcon II) and the map after refinement and post-processing shown with the view distribution calculated from 16,190 particles (surface representation at 4 sigma contour level). C2 symmetry was imposed during refinement. **d** The Fourier Shell correlation (FSC) curve after gold-standard refinement of all particles (26,853) [2]. The FSC = 0.143 criterion [3] indicates a resolution of 7.9 Å. **e** Local resolution of the final TORC2 cryo-EM map from the combined data set shown in palm (above) and dorsal (below) views, calculated from ResMap [4]. The map is contoured at 7.5 sigma level. The core of the complex is resolved at 5 Å whereas peripheral parts have a lower resolution of ~10-12 Å.

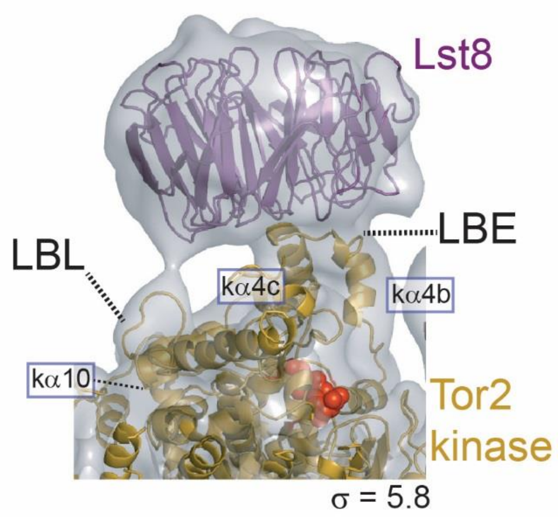

Supplementary Figure 3. Connections between Tor2 and Lst8. Lst8 (purple) is bound to the Tor2 kinase domain (orange-yellow) via a major contact involving the Lst8-binding element (LBE) formed by kinase helices kα4b and kα4c and the connecting loop (nomenclature adapted from Ref. 5). A second connection is formed by the Lst8-binding loop (LBL) between helices kα10 and kα11.

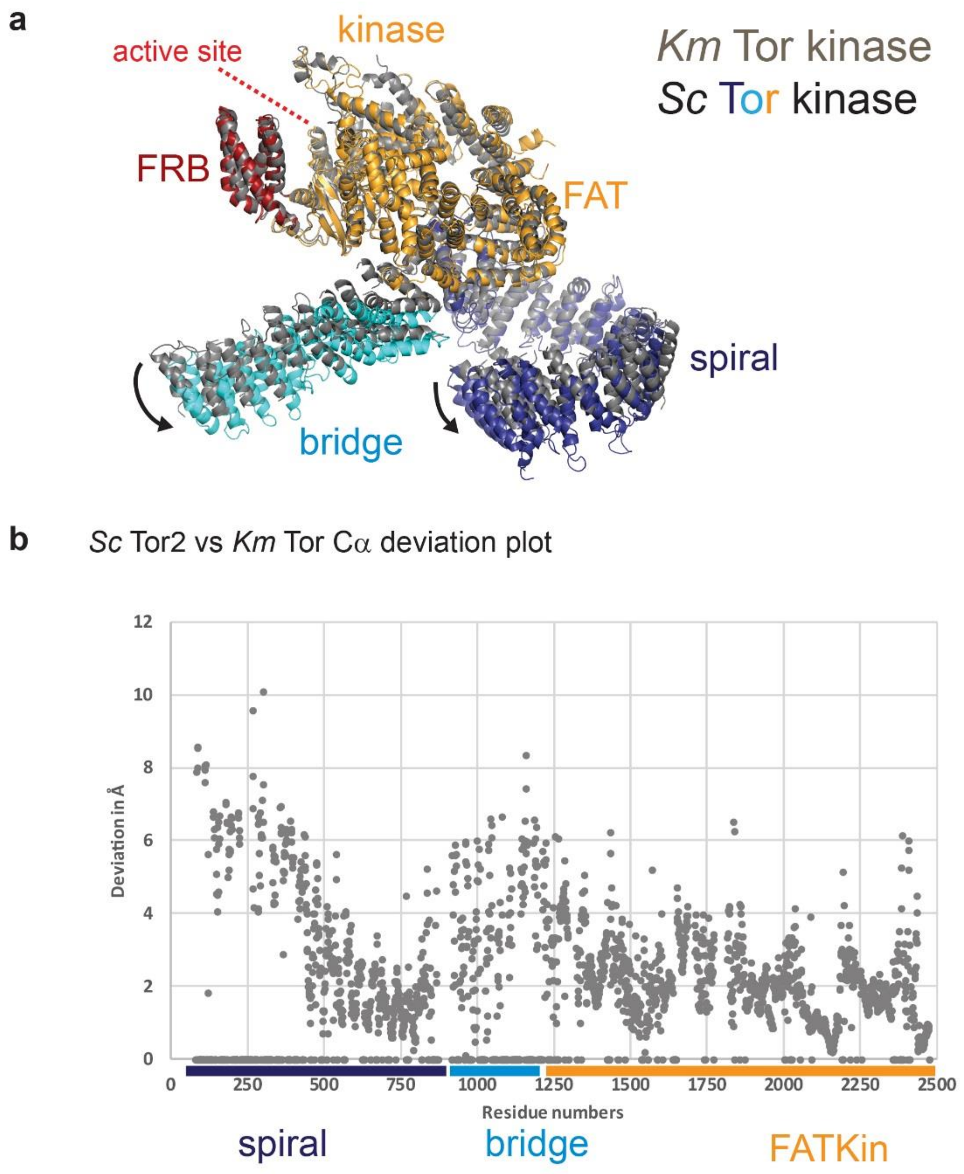

Supplementary Figure 4. Conformation of *S. cerevisiae (Sc)* Tor2 kinase in TORC2 compared to *Km* Tor kinase. a Overlay of the atomic model of *Sc* Tor2 in TORC2 (color coding as in Figure 2) with the atomic model of *Km* Tor (grey) [5]. Arrows indicate conformational changes in the spiral and bridge domains. **b** Plot showing the deviations of the C-alpha residues in the two atomic models (*Sc* Tor2 kinase versus *Km* Tor kinase model), providing a measure for the conformational changes in the N-terminal part of the Tor spiral domain (residues 81-450) and the C-terminal part of the bridge domain (residues 1120-1218). The structures are well-conserved for the FAT and kinase domains (FATKin), with a minimum root-mean-square deviation of 2.4 Å for the C-terminal 918 residues.

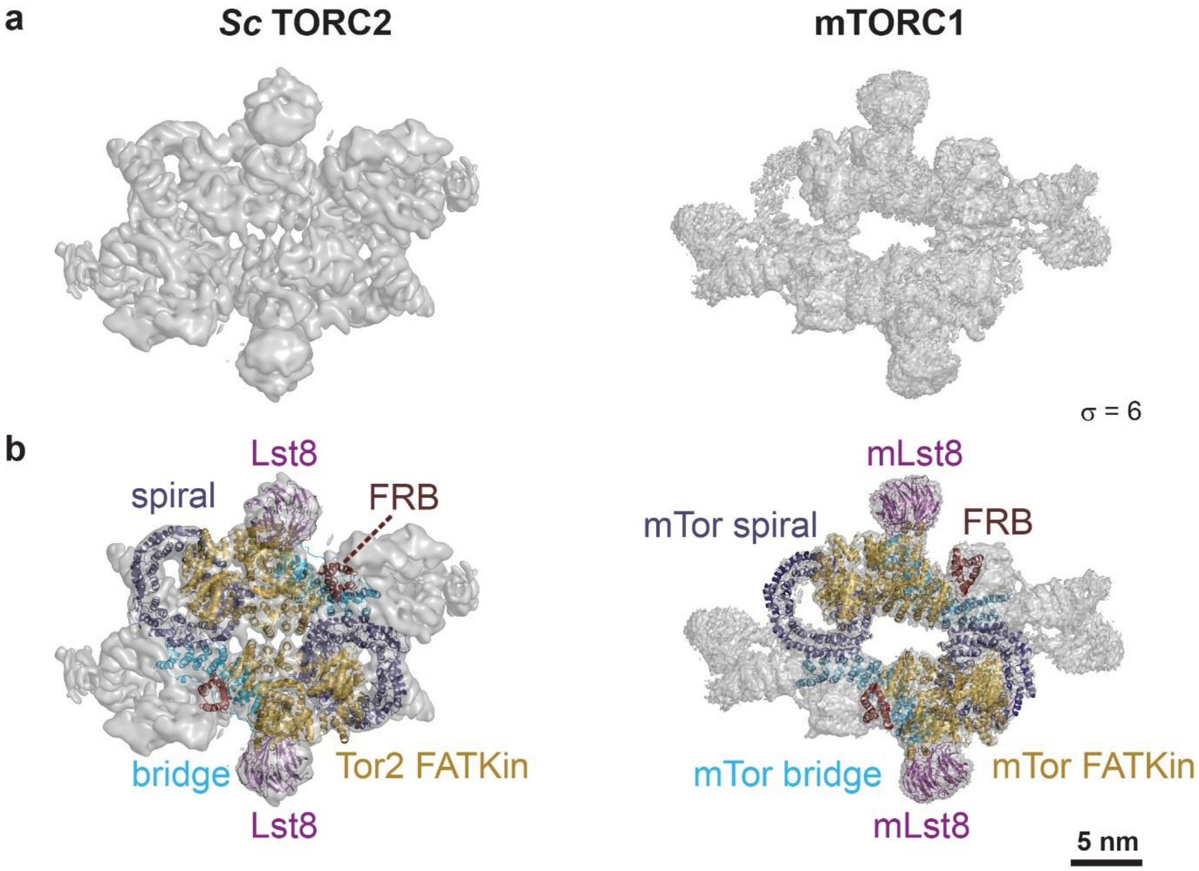

Supplementary Figure 5. Comparison of *S. cerevisiae (Sc)* Tor2 in TORC2 with mTOR in mTORC1. a TORC2 and mTORC1 cryo-EM structures [6] shown at a sigma level of 6 in the same view. **b** Tor2-Lst8 dimer and mTOR-mLst8 fitted into the density of TORC2 and mTORC1 respectively. The spiral is coloured blue, the bridge cyan, the FAT and kinase domain orange-yellow and the FRB domain dark red. Scale bar = 5 nm.

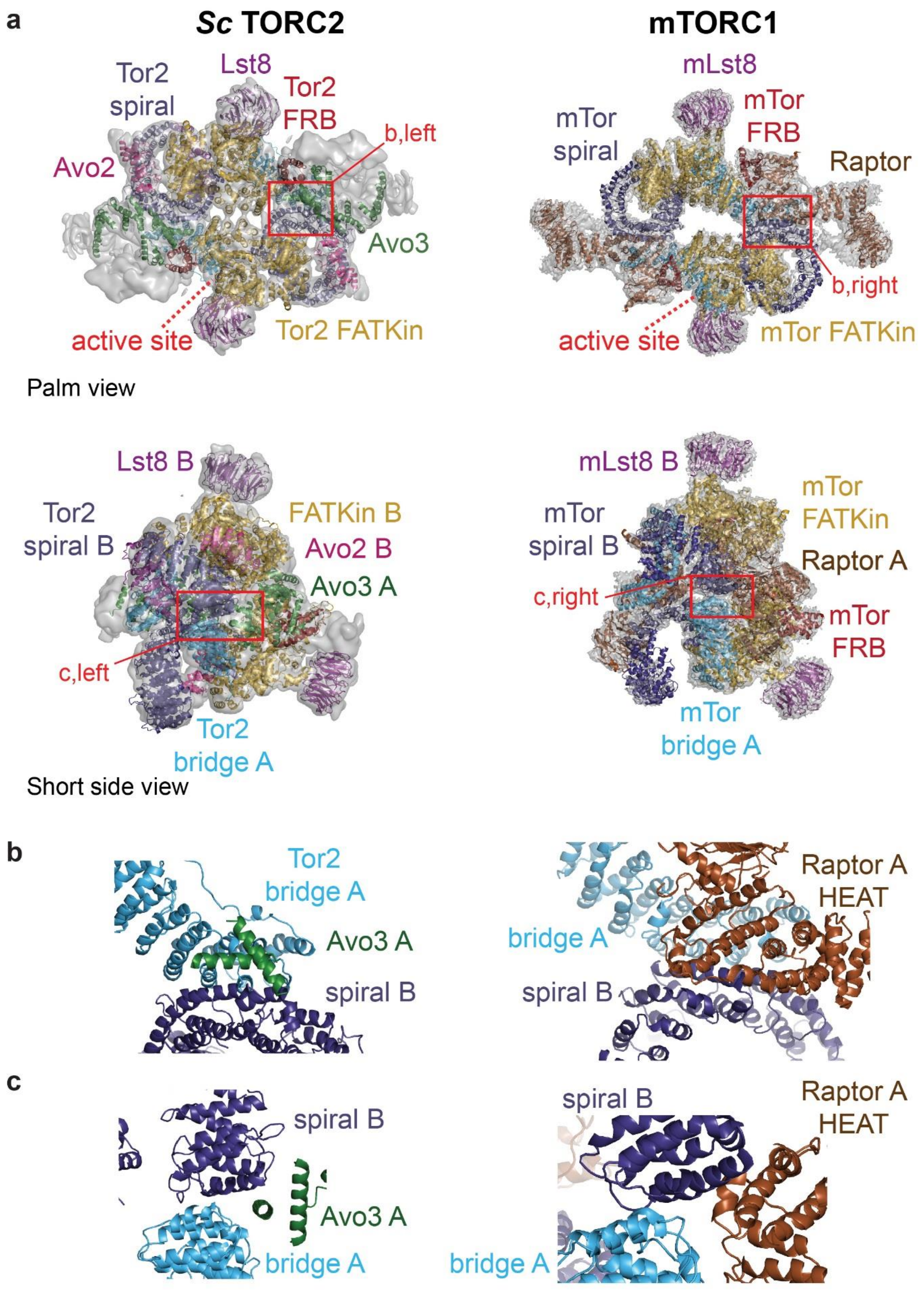

Supplementary Figure 6. Avo3 and Raptor binding to Tor-Lst8 is mutually exclusive. TORC2 has a more recessed active site cleft. a *Sc* TORC2 (left) and mTORC1 [6] (right)

shown from the palm view (above) and a side view (below). The active site is marked in both complexes for shape comparison. Both EM densities (transparent grey) are shown at a contour level of $\sigma = 6$. Tor2 kinase and mTOR kinase have the same colour coding: N-terminal HEAT domain/ spiral in blue, the middle HEAT domain/ bridge in cyan, the FAT and kinase domain in orange-yellow and the FRB domain in dark red. Avo2 is depicted in magenta, Avo3 in green and human Raptor in brown. The red boxes highlight the area shown in a close-up view in panels b and c. **b,c** Close-up views on the dimer interface formed by Tor2 kinase and Avo3 in *Sc* TORC2 and by mTor kinase and Raptor in mTORC1.

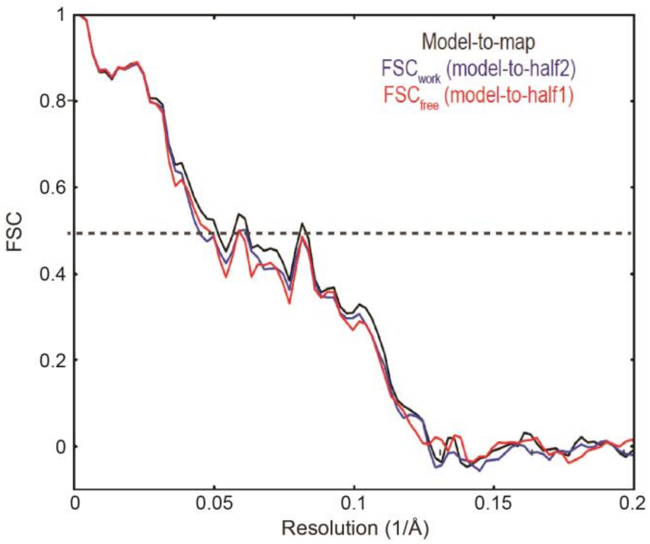

Resolution Limits (Å)	220.000 – 8.000
**Average Fourier shell correlation**	**0.8827**

Supplementary Figure 7. Cross-validation of fitting of the atomic coordinates into the corresponding TORC2 EM map. Right: Half map 2 was used to rigid-body refine against the current partial atomic model in REFMAC [7]. For this refinement, a mask derived from the model applied to half map 2 was used (FSC_{work}). Next, the refined model was compared to half map 1 (FSC_{free}). FSC curves for the self and cross-validation are shown in blue and red respectively, showing no sign of overfitting[7]. The FSC curve in black is calculated between the current partial atomic model and its corresponding part of the final EM map. The average FSC of 0.88 between model and map indicates a good overall quality of the fitting.

Supplementary Table 1. Details of the TORC2 atomic model

TORC2 subunits	Domain	Homology model template	Residues	Model description	Building and fitting
Tor2	Spiral /Horn	5fvm	81-871	Main chain plus C-beta with residues assigned	The model is adjusted to fit into observed helices using COOT
	Bridge	5fvm	916-1296	Main chain plus C-beta with residues assigned	The model is adjusted to fit into observed helices using COOT
	FATKIN	5fvm	1321-2474	Main chain plus C-beta with residues assigned	The model is adjusted to fit into observed helices using COOT
Lst8	WD40	4jsn chainD	3-302	Main chain plus C-beta with residues assigned	The model is fit into density using COOT
Avo1	CRIM	2rvk	647-792	C-alpha trace with residues assigned	The model is fit into density contoured at 3 sigma using COOT
Avo2	N-terminal ANKY-repeats	1n11 chainA	4-163	Main chain plus C-beta with residues assigned	The model is adjusted to fit into observed helices using COOT
Avo3				Poly(Ala) model with main chain plus C-beta	17 individual alpha-helices with an average length of 20 aa, build at the helical density seen at 7.5 sigma contour level

Supplementary Table 2. List of the cross-correlation values for all docked atomic coordinates into the corresponding subunit EM map

The cross-correlation values were determined using Chimera real space fitting [8]. PDBs filtered to 10 Å are compared to the EM density at 7.5; except for the Avo1 CRIM domain model where the corresponding EM density was displayed at 3 sigma.

Subunit	Cross-correlation
Tor2	0.96
Lst8	0.96
Avo2	0.94
Avo3	0.90
Avo1_CRIM	0.85 (3 sigma map)

Supplementary References

1. Gaubitz, C. *et al.* Molecular Basis of the Rapamycin Insensitivity of Target Of Rapamycin Complex 2. *Molecular Cell* **58,** 977–988 (2015).
2. Scheres, S. H. W. RELION: Implementation of a Bayesian approach to cryo-EM structure determination. *Journal of Structural Biology* **180,** 519–530 (2012).
3. Rosenthal, P. B. & Henderson, R. Optimal Determination of Particle Orientation, Absolute Hand, and Contrast Loss in Single-particle Electron Cryomicroscopy. *Journal of Molecular Biology* **333,** 721–745 (2003).
4. Kucukelbir, A., Sigworth, F. J. & Tagare, H. D. Quantifying the local resolution of cryo-EM density maps. *Nature Methods* **11,** 63–65 (2013).
5. Baretić, D., Berndt, A., Ohashi, Y., Johnson, C. M. & Williams, R. L. Tor forms a dimer through an N-terminal helical solenoid with a complex topology. *Nature Communications* **7,** 11016 (2016).
6. Yang, H. *et al.* 4.4 Å Resolution Cryo-EM structure of human mTOR Complex 1. *Protein & Cell* **7,** 878–887 (2016).
7. Brown, A., Long, F,. Nicholls, R. A., Toots, J., Emsley, P. & Murshudov, G. Tools for macromolecular model building and refinement into electron cryo-microscopy reconstructions. *Acta Crystallographica Section D Biological Crystallography.* **71**, 136–153 (2015).
8. Goddard, T. D., Huang, C. C. & Ferrin, T. E. Visualizing density maps with UCSF Chimera. *Journal of Structural Biology* **157,** 281–287 (2007).
