# Supplementary material for: Cryo-EM structure of Saccharomyces cerevisiae target of rapamycin complex 2
Source: Nat Commun. 2017 Nov 23;8:1729. doi: 10.1038/s41467-017-01862-0 (PMC5700991; doi:10.1038/s41467-017-01862-0)
Supplement: Supplementary file 3 — Description of Additional Supplementary Files [file 41467_2017_1862_MOESM3_ESM.pdf]

Descriptions of Additional Supplementary Files

File Name: Supplementary Movie 1
Descriptions: This movie provides a three-dimensional perspective on the fitting of the atomic models for Tor2 (yellow), Lst8 (purple), Avo2 (magenta) and Avo3 (forest green) into the TORC2 reconstruction. The density is segmented according to the subunits (same colour code as atomic models) including Avo1 (green-yellow) and Bit61 (dark red). First, unfilled density is shown in with high transparency, subsequently this unoccupied density is highlighted while the density with atomic coordinates becomes transparent.

File Name: Supplementary Movie 2
Descriptions: This movie shows different views of the *S. cerevisiae* Tor2-Lst8 atomic model (Tor2: yellow; Lst8: purple) and the intra-molecular crosslinks (in red) identified by mass spectrometry[1]. Next, the segmented density corresponding to Avo1 (green-yellow), Avo3 (forest green) and Bit61 (dark red) is added and inter-molecular crosslinks between Lst8 and Avo1, Tor2 and Avo1, Tor2 and Avo3, and finally Tor2 and Bit61 are displayed. The red dotted spheres are centred on the localization of the crosslinking lysines in the Tor2-Lst8 model. The sphere radius of 19.3 Å corresponds the average distance for intra-molecular crosslinks in Lst8 and Tor2 in our atomic Tor2-Lst8 model. For clarity, we do not show the maximal crosslinking distance which is ~30 Å. Avo1 density is displayed at 3 sigma; Avo3 and Bit61 densities at 7.5 sigma contour level.
