# Supplementary material for: Cryo-EM structure of Saccharomyces cerevisiae target of rapamycin complex 2
Source: Nat Commun. 2017 Nov 23;8:1729. doi: 10.1038/s41467-017-01862-0 (PMC5700991; doi:10.1038/s41467-017-01862-0)
Supplement: Supplementary file 2 — Peer Review File [file 41467_2017_1862_MOESM2_ESM.pdf]

PEER REVIEW FILE

Reviewers' comments:

Reviewer #1 (Remarks to the Author):

The manuscript of Schaffitzel and her colleagues represents an important new step in understanding the TORC2 complex. While much is known about the structure and regulation of TORC1, quite a bet less is known about TORC2. The manuscript describes a model for the TOR2 polypeptide in the context of TORC. It also shows much of the architecture of Avo3 (RICTOR). Most importantly, the work makes clear at a structural level why TORC2 is not inhibited by rapamycin and why the TORC1 and TORC2 complexes are mutually exclusive, due to the RAPTOR and RICTOR subunits occupying similar places. This is an important steep forward and will be of a broad interest. The structure is technically well done, and the authors are measured in their interpretations. As with other PIKK structures that have been reported, the local resolution varies greatly, and the authors have appropriately tailored their description of the structure taking this into account. Supplementary Table 1 is a particularly good summary of the level of detail for each subunit/domain in the model.

The location of Avo1 in this cryo-EM map is one of the most poorly defined characteristics of the complex. The earlier work had suggested that this is in the density that corresponds to the Lst8 subunit. The current interpretation is that Avo1 is poorly resolved but at least part of it is near the active site and in contact with the Lst8. This explanation is feasible and is consistent with the negative-stain observations and the cross-linking data.

An important point that the authors make is that the occlusion of the FRB by Avo3 indicates that the FRB cannot be a substrate-binding site for TORC2. This observation suggests re-examination of the role of the FRB in substrate and phosphatidic acid binding.

There are a few minor points that should be addressed.

On lines 74 and 75 on p. 4, the authors quote Knutson (2009, ref 31), saying that the kinase domain is flanked by the FRB domain and the FATC domain. The Knutson paper was published before any structural work on the PIKKs. The detailed structure of the mTOR FATKIN published in 2014 by Pavletich and colleagues showed that the FRB can be regarded as an insertion in the N-lobe of the kinase domain. This insertion occurs just after kalpha1 of the kinase domain, which is analogous to the first helix in the kinase domain of the PI3Ks. It would

be best to rephrase this to say that the FRB is an insertion in the kinase domain. Indeed, this is what is illustrated by the authors in Fig 1A.

On p. 7, lines 144-145. The authors state "… the spiral and bridge domains of Tor2 form the majority of the protomer-protomer interface in TORC2 and also in mTORC132,34." This statement about mTORC1 is not accurate for reference 32. In reference 32, the contact referred to was proposed to be within one protomer, not a protomer-protomer interface. Reference 34 describes this as a protomer-protomer interface, using the revised topology as described in references 33 and 34.

Similarly, on lines 181-182, p. 8, the sentence "Notably, in human mTORC1 the central armadillo domain of Raptor similarly contacts the bridge of mTOR in one protomer and the spiral of mTOR in the second protomer32,32" is accurate only for reference 34. In reference 32, the Raptor was proposed to bridge bridge and spiral in the same protomer.

In the discussion, the authors propose "We note that our placement of the PH domain of Avo1 above the WD40 repeats of Lst8 is analogous to the recently reported structure of Vps34 complex. In this complex, the WD40 repeats of Vps15 support the membrane binding BARA domain of Vps30 and Vps38." In the Vps34 complex, the side of the Vps15 WD40 domain contacts a BARA domain. The top of the Vps15 WD40 domain contacts a coiled-coil that does not appear to be involved in membrane binding. It would appear that this is the surface analogous to where the authors place Avo1. WD40 domains are capable of making interactions in a variety of ways, involving top, bottom and sides of the domains, depending on the domain and binding partner.

Reviewer #2 (Remarks to the Author):

The authors previously reported a structure model of budding yeast TOR complex 2 (TORC2) based on data from crosslinking-mass spectrometry and EM (Gaubitz et al. 2015, cited) experiments. The current manuscript also reports the EM structure of budding yeast TORC2 but at a higher resolution with the Titan Krios EM. The authors have attempted to decipher the subunit architecture of TORC2, by fitting to their EM data the available crystal, NMR and EM structures of TOR, LST8 and AVO1 as well as the predicted alpha-helical domain structures of AVO2 and AVO3.

With their EM images at a higher resolution and recent results from other laboratories, the

authors now propose subunit arrangements significantly different from those presented in Gaubitz et al. (2015). Despite the best practice used in assigning the individual subunits within the TORC2 structure, the credibility of the new architecture model seems to be limited without additional experimental confirmations. Other issues are summarized below.

1. lns 27-29, “Avo3 additionally binds the FKBP12-rapamycin-binding site of the Tor2 FRB domain. This interaction renders TORC2 insensitive to rapamycin….”
This result is not novel, as a similar conclusion had already been reported by the authors (Gaubitz et al., 2015).

2. lns 209-210, “We propose that the density on top of Lst8 corresponds to the PH domain of Avo1 which tethers TORC2 to the membrane.”
The authors should test whether the “Avo1 density” in Fig. 4A is indeed derived from the PH domain of Avo1, with the Avo1 ΔPH mutant they used in Gaubitz et al. (2015).

3. Fig. 4D
The exact position where GFP was inserted in Avo1 should be described. In addition, the authors should provide firm evidence that the additional density observed by the GFP insertion (asterisks in Fig. 4D) is indeed derived from GFP itself, rather than from an altered conformation of Avo1 and/or other subunits.

Minor points:

ln 142: “part of the spiral domain and residues and in the bridge domain” should be “part of the spiral domain and residues in the bridge domain”?

Supplementary Figure 1C: “Finger3B” and “Finger4B” in light blue should be “Finger3A” and “Finger4A”, respectively?

Reviewer #3 (Remarks to the Author):

In this article, Karuppasamy and colleagues report the sub-nanometer resolution cryo-EM structure of yeast TORC2. The authors find that the Avo3/Rictor subunit (unique to TORC2) bind to the same site on Tor2 as the Kog1/Raptor subunit in the mammalian counterpart complex, explaining why these two factors are mutually exclusive. The structure also explains why TORC2 is insensitive to rapamycin: subunit Avo3 binds to the FKBP12-rapamycin-binding site. The authors find that Avo1/hSin1 is mobile however claim that the CRIM domain maps in

proximity to the active-site-cleft of TORC2.

This manuscript is very well written and the authors do a great job at describing a complicated structure in a simple, easy to follow manner. The 3D reconstruction work is done carefully. The presentation of the atomic model however should be improved and the subunit tagging work should be presented differently. Overall this study advances our understanding of TOR-containing complexes, although the major elements of novelty appear to lie in the region of the structure that is unfortunately less-well defined/interpretable.

Here are specific points should be addressed before publication.

1. Supplementary figure 1 panel A, some of the members of the TORC2 complex appear to be extensively proteolysed. How much does this proteolysis affect the structure presented?

2. Supplementary Figure 1 panel C. Fingers and thumb are defined here and this nomenclature is used in the main text. This nomenclature is however absent in the main figures, making them a bit hard to follow.

3. Given the complexity of this structure, it is difficult for this reviewer to evaluate the quality of the atomic model, apart from the higher resolution, mainly helical core of this structure. It would be good to have a movie with segmented density for all of the modelled subunits, with the docked atomic coordinates. It would then be good to display the unoccupied density as well as a separate entity in the movie. For all docked atomic coordinates, it would be good to give a cross-correlation score - or FSC - (comparing the preferred solution with second best docking solution).

4. This work builds on very elegant work published by the same authors that used negative stain EM combined with XL/MS. It would be good to use the available XL/MS data more, to visually explain how the integrated use of the higher resolution cryo-EM and the structural mass spectrometry can be employed to generate a reliable molecular model.

5. It would be good to have an end-on view of the WD40 domain of Lst8. Do they contain a cavity at the centre of the'doughnut', which would be expected at this resolution?

6. Figure 4 panel B. I don't think that selectively showing the structure of Avo1 at a different contour level is a good idea. The authors should show the whole structure at sigma=3 (alongside sigma=7.5). Is there additional density departing from other regions in the structure, which could have been interpreted as meaningful density?

7. Figure 4 panel D The additional density marked with an asterisk by the authors does not seem

to be the only difference between the untagged control and the internal GFP-fusion protein. This limitation, together with my concerns summarised in point 6, pose some questions about the assignment/modelling of the Avo1 CRIM domain.

8. Results, page 6. "The final reconstruction yielded a rhombohedral shape with 2-fold rotational symmetry." This sentence is poorly written, given that 2-fold rotational symmetry was imposed during the reconstruction.

9. Number of particles. In the results it is claimed: "the initial data set comprised 111,022 particles of which 16,190 were included in the finar reconstruction." In the methods an additional 10,663 particles are mentioned, which were required to reach a final resolution of 7.9A. Does the number of particles in the results need to be revised?

Point-by-point responses to reviewers' comments:

Reviewer #1 (Remarks to the Author):

The manuscript of Schaffitzel and her colleagues represents an important new step in understanding the TORC2 complex. While much is known about the structure and regulation of TORC1, quite a bet less is known about TORC2. The manuscript describes a model for the TOR2 polypeptide in the context of TORC. It also shows much of the architecture of Avo3 (RICTOR). Most importantly, the work makes clear at a structural level why TORC2 is not inhibited by rapamycin and why the TORC1 and TORC2 complexes are mutually exclusive, due to the RAPTOR and RICTOR subunits occupying similar places. This is an important step forward and will be of a broad interest. The structure is technically well done, and the authors are measured in their interpretations. As with other PIKK structures that have been reported, the local resolution varies greatly, and the authors have appropriately tailored their description of the structure taking this into account. Supplementary Table 1 is a particularly good summary of the level of detail for each subunit/domain in the model.

The location of Avo1 in this cryo-EM map is one of the most poorly defined characteristics of the complex. The earlier work had suggested that this is in the density that corresponds to the Lst8 subunit. The current interpretation is that Avo1 is poorly resolved but at least part of it is near the active site and in contact with the Lst8. This explanation is feasible and is consistent with the negative-stain observations and the cross-linking data.

An important point that the authors make is that the occlusion of the FRB by Avo3 indicates that the FRB cannot be a substrate-binding site for TORC2. This observation suggests re-examination of the role of the FRB in substrate and phosphatidic acid binding.

We thank this referee for the positive assessment of our work and the helpful comments to improve the manuscript.

There are a few minor points that should be addressed.

On lines 74 and 75 on p. 4, the authors quote Knutson (2009, ref 31), saying that the kinase domain is flanked by the FRB domain and the FATC domain. The Knutson paper was published before any structural work on the PIKKs. The detailed structure of the mTOR FATKIN published in 2014 by Pavletich and colleagues showed that the FRB can be regarded as an insertion in the N-lobe of the kinase domain. This insertion occurs just after kalpha1 of the kinase domain, which is analogous to the first helix in the kinase domain of the PI3Ks. It would be best to rephrase this to say that the FRB is an insertion in the kinase domain. Indeed, this is what is illustrated by the authors in Fig 1A.

As suggested by the referee, we have changed the sentence to state that the FRB domain is an

insertion and cited the Pavletich structure in addition to Knutson, 2009:

> From N-terminus to C-terminus, Tor contains two blocks of HEAT (Huntingtin, EF3A, ATM, TOR) repeats, a block of TPR (tetratricopeptide) repeats known as the FAT (Frap, ATM, TRRAP) domain, and the kinase domain, which includes the FRB domain inserted in its N-terminal part and the FAT C-terminal (FATC) domain [19,31] (Fig. 1A).

On p. 7, lines 144-145. The authors state "... the spiral and bridge domains of Tor2 form the majority of the protomer-protomer interface in TORC2 and also in mTORC132,34." This statement about mTORC1 is not accurate for reference 32. In reference 32, the contact referred to was proposed to be within one protomer, not a protomer-protomer interface. Reference 34 describes this as a protomer-protomer interface, using the revised topology as described in references 33 and 34.

Thank you for pointing this out: we corrected this, and refer to references 33 and 34 in the revised version of the manuscript:

> Moreover, the spiral and bridge domains of Tor2 form the majority of the protomer-protomer interface in TORC2, the Tor-Lst8 complex and also in mTORC1 [33,34].

Similarly, on lines 181-182, p. 8, the sentence "Notably, in human mTORC1 the central armadillo domain of Raptor similarly contacts the bridge of mTOR in one protomer and the spiral of mTOR in the second protomer32,34" is accurate only for reference 34. In reference 32, the Raptor was proposed to bridge bridge and spiral in the same protomer.

In the revised manuscript, we have deleted reference 32 and now refer only to reference 34.

In the discussion, the authors propose "We note that our placement of the PH domain of Avo1 above the WD40 repeats of Lst8 is analogous to the recently reported structure of Vps34 complex. In this complex, the WD40 repeats of Vps15 support the membrane binding BARA domain of Vps30 and Vps38." In the Vps34 complex, the side of the Vps15 WD40 domain contacts a BARA domain. The top of the Vps15 WD40 domain contacts a coiled-coil that does not appear to be involved in membrane binding. It would appear that this is the surface analogous to where the authors place Avo1. WD40 domains are capable of making interactions in a variety of ways, involving top, bottom and sides of the domains, depending on the domain and binding partner.

We agree with the referee that WD40 domains coordinate protein-protein interactions in many different ways. In the revised version of the manuscript, we have therefore deleted the part referring to interactions of the Vps15 WD40 repeat with Vps30 and Vps38.

Reviewer #2 (Remarks to the Author):

The authors previously reported a structure model of budding yeast TOR complex 2 (TORC2) based on data from crosslinking-mass spectrometry and EM (Gaubitz et al. 2015, cited) experiments. The current manuscript also reports the EM structure of budding yeast TORC2 but at a higher resolution with the Titan Krios EM. The authors have attempted to decipher the subunit architecture of TORC2, by fitting to their EM data the available crystal, NMR and EM structures of TOR, LST8 and AVO1 as well as the predicted alpha-helical domain structures of AVO2 and AVO3.

With their EM images at a higher resolution and recent results from other laboratories, the authors now propose subunit arrangements significantly different from those presented in Gaubitz et al. (2015). Despite the best practice used in assigning the individual subunits within the TORC2 structure, the credibility of the new architecture model seems to be limited without additional experimental confirmations. Other issues are summarized below.

We respectfully disagree with this referee and wish to clarify:

1. Our previous study (Gaubitz et al., Mol Cell 2015) reported the first description of the architecture of TORC2 using negative stain EM and crosslinking - mass spectrometry (XL-MS). The negative stain EM reconstruction had a resolution of 26 Å and we could only tentatively assign TORC2 subunits to discernible features of the EM map. Here, we report the first cryo-EM structure of a TORC2 complex at a resolution of 5-7 Å in the central parts of the complex. In summary, we present here a state-of-the-art cryo-EM structure, the first one for the TOR complex 2, which is much better resolved (up to 20 Å !) than the previous negative stain reconstruction.

2. In our current manuscript, we fit atomic models only into the central part of TORC2 which is resolved to 5-7 Å. For this central part secondary structure elements are clearly resolved.

3. The referee states that the subunit arrangements in the high-resolution structure significantly differs from those previously presented. We disagree with this statement. Our new TORC2 subunit arrangements are actually in <u>good</u> agreement with our <u>previous tentative</u> localization which was based on bulky tags, antibodies or subunit deletion/truncation and negative stain EM and on crosslinking-mass spectrometry (Gaubitz et al., 2015). Notably, we had correctly located the kinase active site of Tor2 in the 26 Å TORC2 map, and the subunits Avo2 and Avo3. Moreover, the Tor-Lst8 arrangement in TORC2 agrees very well with the recent cryo-EM structures of the yeast Tor-Lst8 dimer (Baretic et al., 2016) and mTor-mLst8 arrangement in mTORC1 (Yang et al., 2016). This indicates that the structural core of TORC1 and TORC2 is formed by the same Tor-Lst8 dimer. The location of Lst8 and Avo1 in TORC2 had to be re-interpreted; in our previous model we made it very clear in our prior text that these were tentative placements and fittings as our analyses

of TORC2 lacking Lst8 did not reveal a discrete density loss consistent with a WD40 propeller (Gaubitz et al., 2015). In our cryo-EM structure, the well-resolved density in the 'Thumb' is clearly occupied by the WD40 repeats of Lst8 (see below, answer to Referee3), and our new interpretation is consistent with our previous negative-stain localizations and the cross-linking data.

Taken together, it remains unclear to us why this referee questions the assignment of subunits in TORC2 and which additional experimental confirmations he/she would expect to see - we already performed XL-MS and negative stain EM localization experiments and re-visit these data in light of the present higher resolution structure. In the revised manuscript, we additionally provide a FSC curve comparing atomic model and map as well as a list of cross correlation values for the docked atomic coordinates, highlighting the good agreement of data and model (see below, answer to Referee3).

1. lns 27-29, "Avo3 additionally binds the FKBP12-rapamycin-binding site of the Tor2 FRB domain. This interaction renders TORC2 insensitive to rapamycin...."
This result is not novel, as a similar conclusion had already been reported by the authors (Gaubitz et al., 2015).

We disagree with this statement:
In our previous study, we found that deletion of the C-terminal part of Avo3 renders TORC2 rapamycin-sensitive using a yeast genetic screen. Accordingly, we suggested that binding of the C-terminal part of Avo3 to the FRB domain *interferes* with FKBP12-rapamycin binding in TORC2.
Here, we visualize at high-resolution why the TOR complex 2 is insensitive to rapamycin. The fact that a folded domain of Avo3 directly *occupies* the FKBP12-rapamycin-binding site of the FRB domain has important functional implications: the FRB cannot serve as a substrate or phosphatidic acid binding site in TORC2 as previously suggested.
Taken together, we confirm here the results of our previous genetic study and report the *structural basis of the rapamycin-insensitivity* of TORC2.

2. lns 209-210, "We propose that the density on top of Lst8 corresponds to the PH domain of Avo1 which tethers TORC2 to the membrane."
The authors should test whether the "Avo1 density" in Fig. 4A is indeed derived from the PH domain of Avo1, with the Avo1 ΔPH mutant they used in Gaubitz et al. (2015).

We realize now that our wording was misleading. To avoid misunderstandings, we have changed the text as follows:

> Based on these and our previous localization efforts with negative stain EM involving TORC2 preparations in which the PH domain was either deleted or C-terminally tagged and labelled

with an antibody [24], we propose that the density on top of Lst8 corresponds to Avo1, including its PH domain which tethers TORC2 to the membrane [41]

Concerning the test for Avo1 density, several experiments support our proposed localization:

(1) To localize Avo1 we previously used negative stain EM and Avo1 variants. One of the variants lacked the C-terminal PH domain (Avo1 ΔPH). In 2D class averages of negative stained images, density above the Thumb region of TORC2 was less pronounced compared the density observed for TORC2 comprising wildtype Avo1 (see Figure 1, left). It should be noted, however, that the density does not completely disappear. A second Avo1 variant comprised an additional C-terminal myc-tag immediately following the PH domain. Binding of an anti-myc antibody (α-myc) to TORC2 with the Avo1-myc tag variant gives rise to additional density above the Thumb in 2D class averages from negative stained EM images (Figure 1, right).

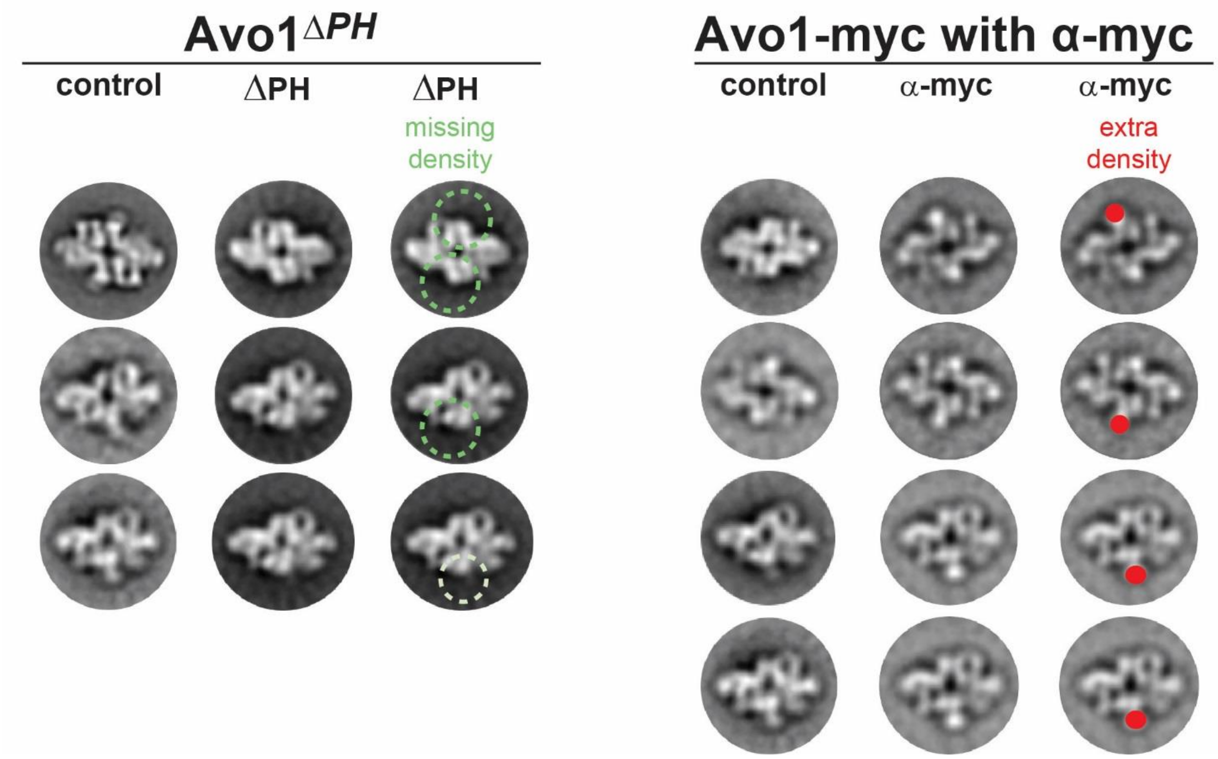

Rebuttal Figure 1, adapted from Gaubitz et al. 2015: Negative stain 2D EM analysis of TORC2. Left: Deletion of the Pleckstrin Homology (PH) domain of Avo1 resulted in dampened electron density next the Thumb (dotted pale green circle). Right: Avo1 with a C-terminal myc-tag was localized using an anti-myc antibody which could be detected as additional density next to the Thumb (stained in red).

(2) Furthermore, XL-MS data support the notion that Avo1 is very flexible and close to Tor2-Lst8 (Figure 2, and Supplementary Movie 2 of our manuscript). Specifically, we have a cluster of diagnostic crosslinks in Lst8 and the Tor2 kinase domain, but unless the contour level is dropped there is no adjacent unassigned density in the cryo-EM map which could accommodate a 131 kDa protein (see Figure 5 of our manuscript) or into which we could fit the Avo1 PH domain structure.

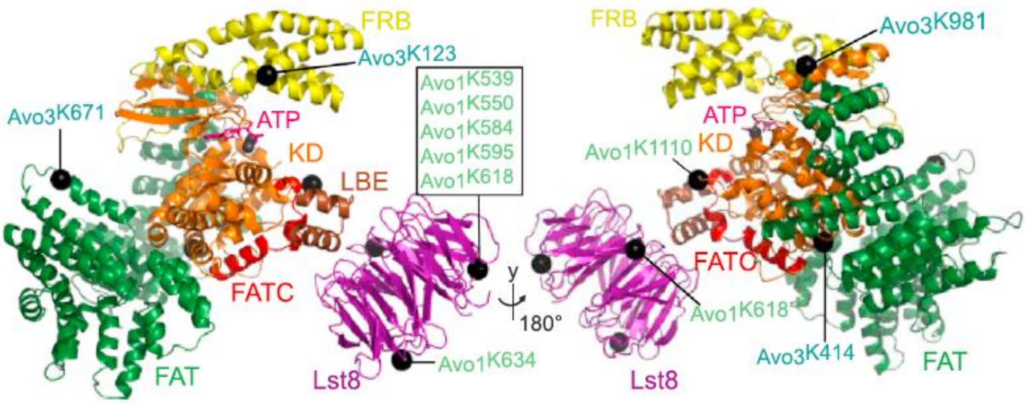

Rebuttal Figure 2, taken from Gaubitz et al. 2015: Schematic representation of Avo1 and Avo3 crosslinks with the Tor2ΔN-Lst8 model. The Tor2 FAT domain is depicted in green, the FRB domain in yellow, the catalytic domain in orange, the Lst8-binding element in brown, and the FATC domain in red. Lysine residues involved in crosslinks are represented as black spheres.

Having clarified in the revised manuscript that the diffuse cryo-EM density is attributed to Avo1 - and *not exclusively* to the Avo1 PH domain - we are not sure which additional insights could be provided by using the Avo1 ΔPH construct beyond what we have shown by negative stain EM already.

3. Fig. 4D: The exact position where GFP was inserted in Avo1 should be described. In addition, the authors should provide firm evidence that the additional density observed by the GFP insertion (asterisks in Fig. 4D) is indeed derived from GFP itself, rather than from an altered conformation of Avo1 and/or other subunits.

GFP was inserted C-terminally to the CRIM domain (residues 647-792), after residue 794. We have included this information in the Figure Legend of Figure 4.

As requested, we provide additional negative stain 2D class averages of TORC2 and of the TORC2 variant with Avo1-CRIM-GFP insertion. The data support our localization of the Avo1 CRIM domain close to the active site. We include these reference-free negative stain 2D class averages as Figure 4, panel D in the revised version of the manuscript.

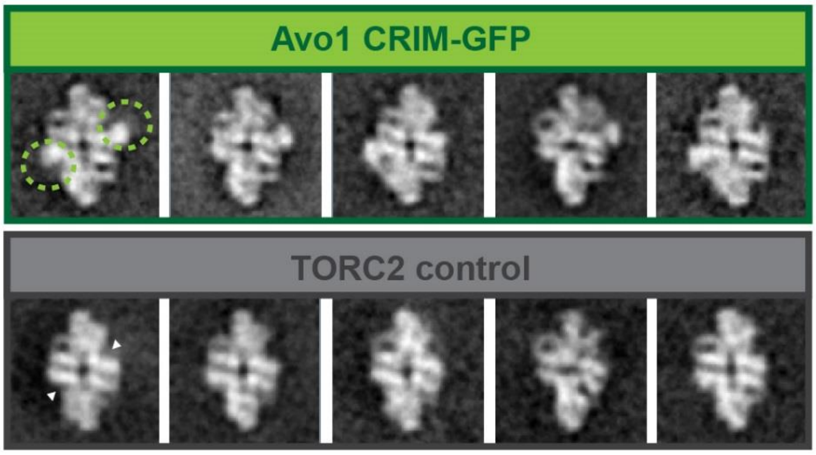

Figure 4, panel D: Insertion of GFP into Avo1 after residue 794, immediately C-terminal to the CRIM domain (residues 647-792), generates new density inside the active site cleft in negative stain 2D class averages. New density is highlighted by green circles in the panel above; the active site of TORC2 is marked by white triangles below.

Moreover, the position of the CRIM domain adjacent to Lst8 and close to the active site is corroborated by the XL-MS data between Lst8 and the middle part of Avo1 involving residues adjacent to the CRIM domain (see Figure 2 above, and manuscript Supplementary Movie 2).

Lastly, although we are still confident in our placement of the CRIM domain, in the revised manuscript we have toned down this conclusion with the inclusion of the following phrase:

> "Although we cannot rule out that this new density represents a displacement of a native TORC2 subunit, collectively, these observations suggest that Avo1 localizes to the length and the tip of the Thumb, in proximity to the active site cleft, but adopts multiple conformations limiting the resolution of this region."

Minor points:

ln 142: "part of the spiral domain and residues and in the bridge domain" should be "part of the spiral domain and residues in the bridge domain"?

Thank you. We have corrected this in the revised version of the manuscript.

Supplementary Figure 1C: "Finger3B" and "Finger4B" in light blue should be "Finger3A" and "Finger4A", respectively?

We have changed **Supplementary Figure 1C** accordingly:

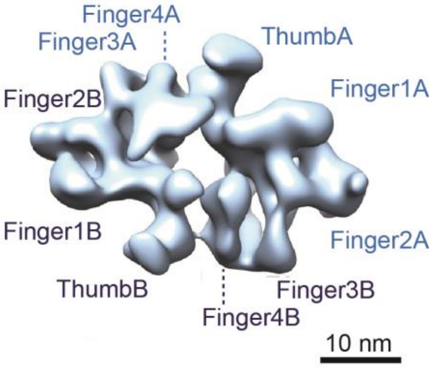

Reviewer #3 (Remarks to the Author):

In this article, Karuppasamy and colleagues report the sub-nanometer resolution cryo-EM structure of yeast TORC2. The authors find that the Avo3/Rictor subunit (unique to TORC2) bind to the same site on Tor2 as the Kog1/Raptor subunit in the mammalian counterpart complex, explaining why these two factors are mutually exclusive. The structure also explains why TORC2 is insensitive to rapamycin: subunit Avo3 binds to the FKBP12-rapamycin-binding site. The authors find that Avo1/hSin1 is mobile however claim that the CRIM domain maps in proximity to the active-site-cleft of TORC2.

This manuscript is very well written and the authors do a great job at describing a complicated structure in a simple, easy to follow manner. The 3D reconstruction work is done carefully. The presentation of the atomic model however should be improved and the subunit tagging work should be presented differently. Overall this study advances our understanding of TOR-containing complexes, although the major elements of novelty appear to lie in the region of the structure that is unfortunately less-well defined/interpretable.

We thank this referee for the overall positive assessment of our work and for the constructive, helpful comments to improve the manuscript.

Here are specific points should be addressed before publication.

1. Supplementary figure 1 panel A, some of the members of the TORC2 complex appear to be extensively proteolysed. How much does this proteolysis affect the structure presented?

We have included a new SYPRO Ruby stained gel showing that the TORC2 subunits are phosphorylated rather than proteolysed and that additional bands are due to co-purification of heat shock proteins (identified by mass spectrometry).

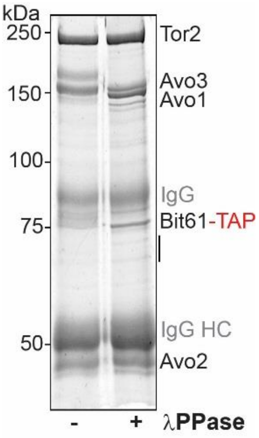

Supplementary Figure 1, panel A. Sypro Ruby-stained SDS PAGE gel of TORC2 after protein-A affinity purification via TAP-tagged Bit61 from cell extracts lacking Bit2. Phosphorylation of TORC2 subunits Avo1 and Bit61 is detected by λ phosphatase treatment. Mass spectrometry additionally detected the presence of heat shock proteins SSA1 (69.6 kDa), SSA2 (69.4 kDa), SSB1 (66.6 kDa) and SSB2 (66.6 kDa), marked with a vertical black line. IgG antibody bands (partially reduced) are labelled.

2. Supplementary Figure 1 panel C. Fingers and thumb are defined here and this nomenclature is used in the main text. This nomenclature is however absent in the main figures, making them a bit hard to follow.

To help the reader, we have included labels for Thumb and Fingers in Figure 1B (palm view) in the revised version of the manuscript.

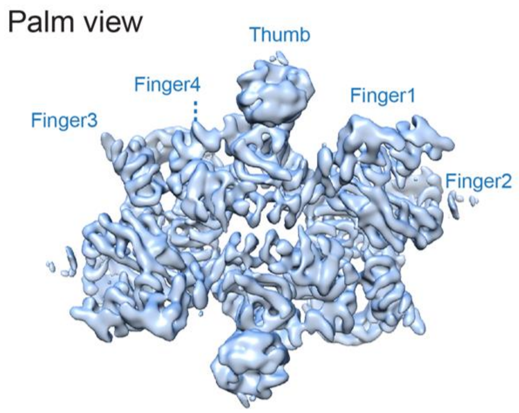

Figure 1, panel B. Cryo-EM structure of TORC2 at an overall resolution of 7.9 Å (FSC criterion 0.143) is shown in a surface representation at 7.5 sigma contour level in four different views. In the palm view, TORC2 lobes are annotated as Thumb and Fingers (1 to 4) for one protomer.

3. Given the complexity of this structure, it is difficult for this reviewer to evaluate the quality of the atomic model, apart from the higher resolution, mainly helical core of this structure. It would be good to have a movie with segmented density for all of the modelled subunits, with the docked atomic coordinates. It would then be good to display the unoccupied density as well as a separate entity in the movie. For all docked atomic coordinates, it would be good to give a cross-correlation score - or FSC - (comparing the preferred solution with second best docking solution).

(1) As suggested, we provide a movie (Supplementary Movie 1) showing the segmented density and the modelled atomic coordinates to help the reader evaluating the quality of the cryo-EM map and the fitting.

(2) We provide a list of the cross-correlation values for all docked atomic coordinates into the corresponding subunit EM map as Supplementary Table 2:

The cross-correlation values were determined using Chimera real space fitting (Goddard et al., 2007). PDBs filtered to 10 Å are compared to the EM density at sigma 7.5; except for the Avo1 CRIM domain model where the corresponding EM density was displayed at 3 sigma level.

**Subunit**	**Cross-correlation**
Tor2	0.96
Lst8	0.96
Avo2	0.94
Avo3	0.90
Avo1_CRIM	0.85 3 sigma)

(3) We calculated the Fourier Shell Correlation between the atomic model and the corresponding parts of the TORC2 map and the half maps using REFMAC (Brown et al., 2015). The average FSC is 0.88, taking into account all fitted parts and using the following settings:

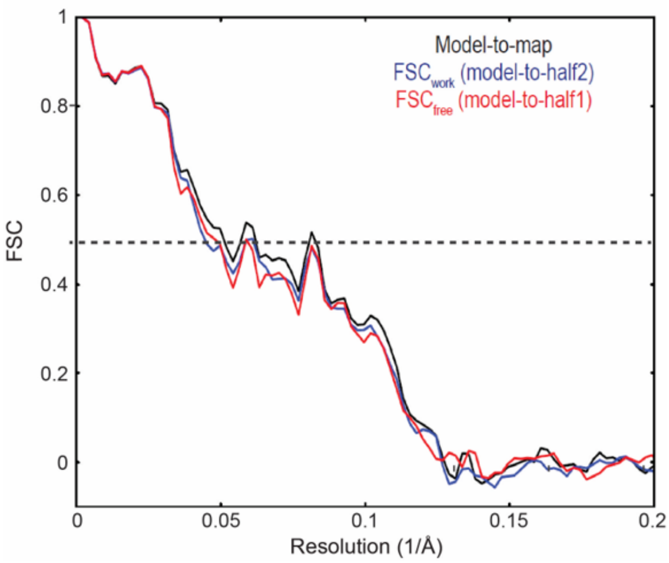

Resolution Limits (Å)	220.000 – 8.000
**Average Fourier shell correlation**	**0.8827**

Supplementary Figure 7. Cross-validation of fitting of the atomic coordinates into the corresponding TORC2 EM map. Right: Half map 2 was used to rigid-body refine against the current partial atomic model in REFMAC [7]. For this refinement, a mask derived from the model applied to half map 2 was used (FSC_{work}). Next, the refined model was compared to half map 1 (FSC_{free}). FSC curves for the self and cross-validation are shown in blue and red respectively, showing no sign of overfitting[7]. The FSC curve in black is calculated between the current partial atomic model and its corresponding part of the final EM map. The average FSC of 0.88 between model and map indicates a good overall quality of the fitting.

4. This work builds on very elegant work published by the same authors that used negative stain EM combined with XL/MS. It would be good to use the available XL/MS data more, to visually explain how the integrated use of the higher resolution cryo-EM and the structural mass spectrometry can be employed to generate a reliable molecular model.

With the revised manuscript, we provide a movie to show how the XL/MS results agree with the atomic model.

Supplementary Movie 2

This movie shows different views of the *S. cerevisiae* Tor2-Lst8 atomic model (Tor2: yellow; Lst8: purple) and the intra-molecular crosslinks (in red) identified by mass spectrometry [24]. Next, the segmented density corresponding to Avo1 (green-yellow), Avo3 (forest green) and Bit61 (dark red) is added and inter-molecular crosslinks between Lst8 and Avo1, Tor2 and Avo1, Tor2 and Avo3, and finally Tor2 and Bit61 are displayed. The red dotted spheres are centred on the localization of the crosslinking lysines in the Tor2-Lst8 model. The sphere

radius of 19.3 Å corresponds the average distance for intra-molecular crosslinks in Lst8 and Tor2 in our atomic Tor2-Lst8 model. For clarity, we do not show the maximal crosslinking distance which is ~30 Å. Avo1 density is displayed a 3 sigma; Avo3 and Bit61 densities at 7.5 sigma contour level.

5. It would be good to have an end-on view of the WD40 domain of Lst8. Do they contain a cavity at the centre of the 'doughnut', which would be expected at this resolution?

Please see below for a clear end-on view of the WD40 domain of Lst8. The doughnut shape of the EM density corresponding to Lst8 is also evident in the Supplementary Movie 1 of the revised version of the manuscript.

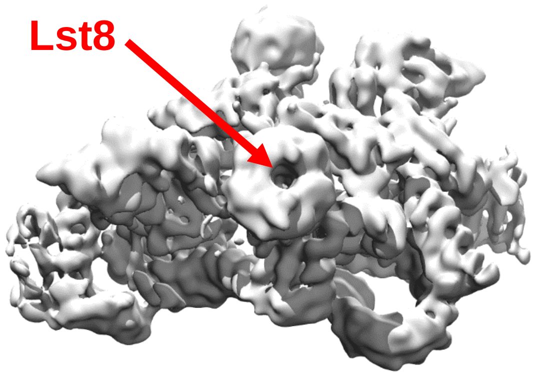

Rebuttal Figure 3. The TORC2 map is contoured in this figure at 6 sigma level. TORC2 is displayed in the long side view such that the doughnut shape of the Lst8 WD40 domain (red arrow) becomes evident.

6. Figure 4 panel B. I don't think that selectively showing the structure of Avo1 at a different contour level is a good idea. The authors should show the whole structure at sigma=3 (alongside sigma=7.5). Is there additional density departing from other regions in the structure, which could have been interpreted as meaningful density?

As suggested, we replaced panel B of Figure 4 with a panel showing TORC2 at a sigma level of 3 next to the same map at sigma level 7.5.
In addition to Avo1, the density attributed to Bit61 becomes pronounced at sigma level 3. High-resolution information regarding Bit61/2 is lacking. Otherwise, we could not identify significant additional density.

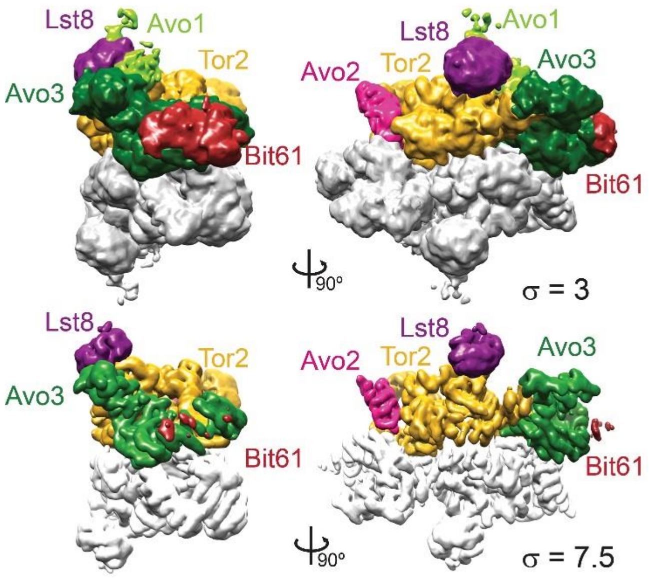

Figure 4, panel B: Surface representations of the TORC2 map in a short side (left) and the palm view (right). Above: TORC2 shown at 3 sigma; below: same views displayed at 7.5 sigma contour level.

7. Figure 4 panel D The additional density marked with an asterisk by the authors does not seem to be the only difference between the untagged control and the internal GFP-fusion protein. This limitation, together with my concerns summarised in point 6, pose some questions about the assignment/modelling of the Avo1 CRIM domain.

To address this comment, we have included additional negative stain 2D class averages comparing TORC2 with TORC2 comprising a Avo1-CRIM-GFP variant as Figure 4, panel D (see above answer to Referee2). The 2D class averages all show extra density next to the kinase active site for the sample containing TORC2 with GFP-labelled CRIM domain.

These data combined with our XL/MS results linking residues adjacent to the CRIM domain to Lst8 further corroborate our conclusion that the CRIM domain of Avo1 is positioned next to Lst8.

Concerning the modelling, we would like to point out that our placement of the CRIM structure is tentative (we state this in the manuscript). We show that this density can accommodate the CRIM domain.

Lastly, although we are confident in our placement of the CRIM domain, in the revised manuscript we have toned down this conclusion (page 10 of the revised manuscript):

> "Although we cannot rule out that this new density represents a displacement of a native TORC2 subunit, collectively, these observations suggest that Avo1 localizes to the length and the tip of the Thumb, in proximity to the active site cleft, but adopts multiple conformations limiting the resolution of this region."

8. Results, page 6. "The final reconstruction yielded a rhombohedral shape with 2-fold rotational symmetry." This sentence is poorly written, given that 2-fold rotational symmetry was imposed during the reconstruction.

Thank you for pointing this out. We modified the sentence as follows:

> The low resolution TORC2 negative stain reconstruction (Supplementary Fig. 1C; [24]) was used as an initial 3D model, yielding a final reconstruction of the TORC2 rhombohedron (Fig. 1B) with an average resolution of 7.9 Å (Supplementary Fig. 2D).

9. Number of particles. In the results it is claimed: "the initial data set comprised 111,022 particles of which 16,190 were included in the final reconstruction." In the methods an additional 10,663 particles are mentioned, which were required to reach a final resolution of 7.9A. Does the number of particles in the results need to be revised?

To clarify this point, we have changed the text on page 6 of the revised version of the manuscript as follows:

> The initial data set comprised 111,022 particles of which 16,190 were included in the final reconstruction (Supplementary Fig. 2B,C). Subsequently, a second data set of 71,519 particles was collected and, after multiple rounds of sorting at 2D and 3D levels, an additional 10,663 particles were included in the final TORC2 reconstruction (26,853 particles in total).

Reviewers' Comments:

Reviewer #1 (Remarks to the Author):

Christianne Berger-Schaffitzel and her colleagues have done an excellent job of responding to the reviewer comments. In particular, the two movies are really helpful for understanding the cross-linking results and the overall arrangements of the subunits. This has added to what was already a excellent contribution. The quantitative analysis of the the model correlation is also a useful addition. This report represents an important work in the field that will be greatly appreciated by the community.

Reviewer #2 (Remarks to the Author):

Revisions and additional clarifications in response to the reviewers' comments have improved the manuscript. However, there remains one reservation in relation to this reviewer's comment #1. The authors' rebuttal to this comment says,
"The fact that a folded domain of Avo3 directly occupies the FKBP12-rapamycin-binding site of the FRB domain has important functional implications: the FRB cannot serve as a substrate or phosphatidic acid binding site in TORC2 as previously suggested. Taken together, we confirm here the results of our previous genetic study and report the structural basis of the rapamycin-insensitivity of TORC2."

On the other hand, the Results section of the manuscript states,
"Due to the lack of a high-resolution Avo3 structure, we are unable to unambiguously assign this density surrounding the FRB domain. However, we found that deletion of the C-terminal 157 amino acids of Avo3 restores access of FKBP12-rapamycin to the FRB domain of Tor2 (ref.24). Therefore, it is reasonable to assume that it is the C-terminal part of Avo3 that contacts the rapamycin-binding site of the FRB domain in the cryo-EM structure."

According to this description in lines 194-198, "a folded domain of Avo3 directly occupies the FKBP12-rapamycin-binding site of the FRB domain" does not seem to be a fact, but a speculation largely based on the authors' previous work (Gaubitz et al., 2015). Thus, this reviewer still feels it is inappropriate to state in the Abstract of the paper, "Avo3 additionally binds the FKBP12-rapamycin-binding site of Tor2's FRB domain rendering TORC2 insensitive to rapamycin and recessing the kinase active-site", as one of the major conclusions of this paper. This statement should be deleted from the Abstract.

Reviewer #3 (Remarks to the Author):

I feel the authors have done an excellent job at addressing my concerns in the revised manuscript. This work is, in my opinion, now ready for publication.

Point-by-point responses reviewer #2's comments:

Reviewer #2 (Remarks to the Author):

Revisions and additional clarifications in response to the reviewers' comments have improved the manuscript. However, there remains one reservation in relation to this reviewer's comment #1. The authors' rebuttal to this comment says "The fact that a folded domain of Avo3 directly occupies the FKBP12-rapamycin-binding site of the FRB domain has important functional implications: the FRB cannot serve as a substrate or phosphatidic acid binding site in TORC2 as previously suggested. Taken together, we confirm here the results of our previous genetic study and report the structural basis of the rapamycin-insensitivity of TORC2."

On the other hand, the Results section of the manuscript states, "Due to the lack of a high-resolution Avo3 structure, we are unable to unambiguously assign this density surrounding the FRB domain. However, we found that deletion of the C-terminal 157 amino acids of Avo3 restores access of FKBP12-rapamycin to the FRB domain of Tor2 (ref.24). Therefore, it is reasonable to assume that it is the C-terminal part of Avo3 that contacts the rapamycin-binding site of the FRB domain in the cryo-EM structure."

According to this description in lines 194-198, "a folded domain of Avo3 directly occupies the FKBP12-rapamycin-binding site of the FRB domain" does not seem to be a fact, but a speculation largely based on the authors' previous work (Gaubitz et al., 2015). Thus, this reviewer still feels it is inappropriate to state in the Abstract of the paper, "Avo3 additionally binds the FKBP12-rapamycin-binding site of Tor2's FRB domain rendering TORC2 insensitive to rapamycin and recessing the kinase active-site", as one of the major conclusions of this paper. This statement should be deleted from the Abstract.

We thank this reviewer for this comment. In fact, we attribute the EM density occluding the FRB domain to Avo3 because we have previously shown in genetic screens that deletions in the C-terminal region of Avo3 render TORC2 rapamycin sensitive.

Accordingly, we have modified our statement in the abstract of the revised manuscript as follows:

> **Abstract.** The Target Of Rapamycin (TOR) kinase assembles into two, distinct multiprotein complexes, conserved across eukaryote evolution. In contrast to TOR Complex1 (TORC1), TORC2 kinase activity is not inhibited by the macrolide rapamycin. Here, we present the structure of *Saccharomyces cerevisiae* TORC2 determined by electron cryo-microscopy. TORC2 contains six subunits assembling into

a 1.4 MDa rhombohedron. Tor2 and Lst8 form the common core of both TOR complexes. Avo3/Rictor is unique to TORC2, but interacts with the same HEAT repeats of Tor2 that are engaged by Kog1/Raptor in mammalian TORC1, explaining the mutual exclusivity of these two proteins. Density, which we conclude is Avo3, occludes the FKBP12-rapamycin-binding site of Tor2's FRB-domain rendering TORC2 rapamycin-insensitive and recessing the kinase active-site. Although mobile, Avo1/hSin1 further restricts access to the active site as its Conserved-Region-In-the-Middle (CRIM) domain is positioned along an edge of the TORC2 active-site-cleft, consistent with a role for CRIM in substrate recruitment.
